# Genetically predicted brain cortical structure mediates the causality between insulin resistance and cognitive impairment

**DOI:** 10.3389/fendo.2024.1443301

**Published:** 2025-01-15

**Authors:** Chaojuan Huang, Yuyang Zhang, Mingxu Li, Qiuju Gong, Siqi Yu, Zhiwei Li, Mengmeng Ren, Xia Zhou, Xiaoqun Zhu, Zhongwu Sun

**Affiliations:** ^1^ Department of Neurology, the First Affiliated Hospital of Anhui Medical University, Hefei, Anhui, China; ^2^ Department of Urology, the First Affiliated Hospital of Anhui Medical University, Hefei, Anhui, China

**Keywords:** brain cortical structure, cognition, insulin resistance, mediation, Mendelian randomization

## Abstract

**Background:**

Insulin resistance is tightly related to cognition; however, the causal association between them remains a matter of debate. Our investigation aims to establish the causal relationship and direction between insulin resistance and cognition, while also quantifying the mediating role of brain cortical structure in this association.

**Methods:**

The publicly available data sources for insulin resistance (fasting insulin, homeostasis model assessment beta-cell function and homeostasis model assessment insulin resistance, proinsulin), brain cortical structure, and cognitive phenotypes (visual memory, reaction time) were obtained from the MAGIC, ENIGMA, and UK Biobank datasets, respectively. We first conducted a bidirectional two-sample Mendelian randomization (MR) analysis to examine the susceptibility of insulin resistance on cognitive phenotypes. Additionally, we applied a two-step MR to assess the mediating role of cortical surficial area and thickness in the pathway from insulin resistance to cognitive impairment. The primary Inverse-variance weighted, accompanied by robust sensitivity analysis, was implemented to explore and verify our findings. The reverse MR analysis was also performed to evaluate the causal effect of cognition on insulin resistance and brain cortical structure.

**Results:**

This study identified genetically determined elevated level of proinsulin increased reaction time (beta=0.03, 95% confidence interval [95%CI]=0.01 to 0.05, *p*=0.005), while decreasing the surface area of rostral middle frontal (beta=-49.28, 95%CI=-86.30 to -12.27, *p*=0.009). The surface area of the rostral middle frontal mediated 20.97% (95%CI=1.44% to 40.49%) of the total effect of proinsulin on reaction time. No evidence of heterogeneity, pleiotropy, or reverse causality was observed.

**Conclusions:**

Briefly, our study noticed that elevated level of insulin resistance adversely affected cognition, with a partial mediation effect through alterations in brain cortical structure.

## 1 Introduction

Epidemiological studies indicate that over 55 million people were affected by dementia in 2019. The World Health Organization projects this number to increase to 139 million by 2050. The economic impact of dementia is expected to escalate from US$1.3 trillion in 2019 to US$2.8 trillion by 2030, presenting significant social and economic challenges (1). The causes of dementia are multifactorial, with Alzheimer’s disease (AD) identified as the primary contributor, accounting for nearly 70% of cases. The cognitive dysfunction associated with dementia is often overlooked in its early stages but progressively worsens, eventually leading to irreversible and incurable conditions in advanced stages. Therefore, this underscores the critical importance of early detection and intervention in managing dementia.

Insulin resistance (IR) is a pathological state characterized by impaired insulin responsiveness, requiring elevated level of insulin to maintain glucose homeostasis in both peripheral tissues and the brain (2), a key feature of Type 2 diabetes mellitus (T2DM) and metabolic syndrome. Additionally, it has been primarily associated with coronary heart disease (3), stroke (4), and AD (5). The hyperinsulinemic-euglycemic clamp, considered the gold standard for measuring insulin resistance, is limited in clinical application owing to its invasiveness, high cost, time-consuming nature, and laborious procedure (6). By comparison, fasting insulin, homeostasis model assessment beta-cell function (HOMA-B), homeostasis model assessment insulin resistance (HOMA-IR), and proinsulin serve as more accessible markers for reflecting insulin resistance (7).

The literature has suggested an association between insulin resistance and cognition. In a previous observational study involving older patients with hypertension, elevated HOMA-IR was related to cognitive impairment (8). Smith et al.’s investigation (9) supported the close relationship between increased HOMA-IR and decreased executive function in patients with vascular cognitive impairment. However, conflicting results from other studies reported no relationship between insulin resistance and AD (10). In a longitudinal study involving older participants without dementia, a higher baseline HOMA-IR was found to predict cognitive degeneration seven years later (11). Despite robust epidemiological evidence, the potential pathogenesis and causal direction between insulin resistance and cognition remain poorly established. Challenges such as selection bias, confounding factors, reverse causality, and relatively small sample size in the observation studies obscure a conclusive resolution to the bidirectional chicken-and-egg question.

Furthermore, limited studies have delved into the underlying mechanisms or mediating pathways connecting insulin resistance and cognition. Previous research has demonstrated alterations in brain cortical structure associated with both insulin resistance (12) and cognitive dysfunction (13). Insulin receptors are extensively expressed in the brain, with predominant distribution in the cerebellum, frontal cortex, and hippocampus, as proved by studies in animal models and post-mortem human brains (14, 15). Thus, insulin may play a crucial role in multiple brain regions. A previous study utilized 18F-fludeoxyglucose - positron emission tomography to measure cerebral glucose metabolism and revealed that blood fasting insulin was linked to glucose metabolism of the inferior parietal, hippocampus, and parahippocampus region (16). Insulin in the peripheral blood might traverse the blood-brain barrier and participate in specific regions’ synaptic and neuronal activity. Various cortical structures serve distinct physiological functions, and cortical atrophy is a recognized pathophysiological process contributing to cognitive impairment. Accordingly, brain cortical structure might be a latent mediator between insulin resistance and cognition.

Mediation analysis (MR) applies single nucleotide polymorphisms (SNPs) closely relevant to the exposure factors as instrumental variables (IVs) to deduce the causality between exposure and outcome (17). Owing to the random assignment of SNPs during meiosis, MR can yield robust causal evidence that is less influenced by confounders and reverse causality. Therefore, MR stands as a well-established statistical method, overcoming limitations inherent in traditional observational studies. Leveraging and extending MR, mediation MR analysis offers an opportunity to assess the complex interlocking causality among insulin resistance, brain cortical structure, and cognition. Moreover, the identified intermediate factors contribute to the exploration of the potential etiology and pathogenesis of cognitive impairment. As far as we know, the causal exploration of mediating pathways from insulin resistance to cognition is lacking. To fill the knowledge gap, our investigation attempted to (i) ascertain whether insulin resistance is causally associated with cognition and (ii) quantify the extent to which brain cortical structure mediates the effects of insulin resistance on cognition.

## 2 Materials and methods

### 2.1 Study design

**Figure 1:**
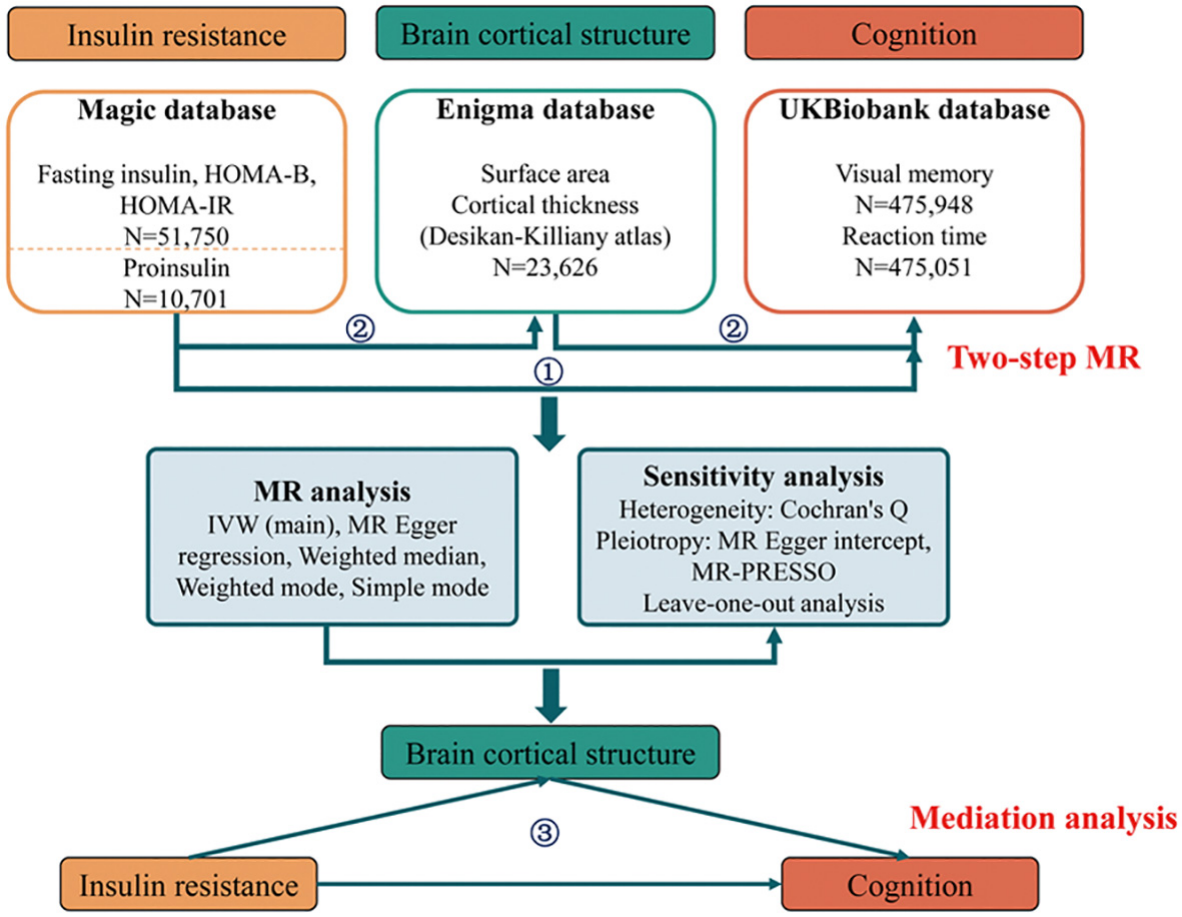
Flowchart of the two-step mediation MR study. MR, Mendelian randomization; HOMA-B, homeostasis model assessment beta-cell function; HOMA-IR, homeostasis model assessment insulin resistance; IVW, inverse-variance weighted; MR-PRESSO, Mendelian randomization pleiotropy residual sum and outlier.

The flowchart in **Figure 1** demonstrates the comprehensive procedure of our exploration. In stage 1, we performed a two-sample bidirectional univariable MR analysis to establish the causality between insulin resistance and cognition. In stage 2, we used a two-step bidirectional univariable MR to select candidate mediators in the causality between insulin resistance and cognition. In stage 3, we constructed a mediation model and quantified the proportion of insulin resistance’s effect on cognition mediated by brain cortical structure. Our study adheres to the STROBE-MR guidelines (**Supplementary Table S1**).

### 2.2 Data sources

#### 2.2.1 Insulin resistance

We used fasting insulin, HOMA-B, HOMA-IR, and proinsulin as established proxies for insulin resistance (1). Towards fasting insulin, HOMA-B, and HOMA-IR (18), we chose genetic IVs from the publicly available meta-analyses of glucose and insulin-related traits consortium (MAGIC), with 51750 participants without diabetes from 26 European cohorts. The three surrogate markers of insulin resistance were log-transformed. The regression analyses were adjusted for age and sex, together with BMI (2). Regarding proinsulin, genome-wide association studies (GWAS) summary statistics were attained from MAGIC either (19). The meta-analysis consisted of 10701 European individuals without diabetes from four cohorts. The regression analyses were adjusted for fasting insulin in addition to age and sex. More detailed characteristics of cohorts have been provided in **Supplementary Table S2**.

#### 2.2.2 Brain cortical structure

Summary statistics for brain cortical structure were derived from the enhancing neuro imaging genetics through meta analysis (ENIGMA) database (20), encompassing 51665 participants across 60 cohorts worldwide. Specifically, 33709 individuals were of European ancestry. Among them, 10803 participants were from the UK Biobank consortium. The imaging phenotype was measured using the T1 structural Magnetic Resonance Imaging sequence combined with the Desikan-Killiany atlas, which contained surficial area (SA) and thickness (TH) for both global and 34 functionally specialized cortical regions. The mean value of global SA was 169647.43 mm^2^, and the mean value of global TH was 2.45 mm. The SA and TH of 34 cortical regions were adjusted based on global measurements to mitigate the impact of individual differences on results. To avoid sample overlap between traits, we employed meta-results involving exclusively European and non-UKB individuals. Consequently, the ultimate sample size used in our study for brain cortical structure was 23626. The detailed cohort information is available in **Supplementary Table S3**.

#### 2.2.3 Cognition

Following existing literature, summary-level statistics for cognition were achieved from the UK Biobank (21), gathering up to 502649 population-based individuals. After excluding patients with neurological disorders, 480416 participants completed the five cognitive assessments through the computerized touchscreen. To magnify statistical power, we chose visual memory and reaction time as proxies for cognition (22). The visual memory was evaluated via a “6 pairs matching” test, requiring individuals to recall and match the position of 6 pairs of cards based on their memory. The number of errors was counted, with higher counts represented poorer cognitive performance. The reaction time was measured through a symbol matching test, akin to a “snap” card game. The completion time (in milliseconds) was recorded, with a longer time symbolized poorer cognitive performance. The scores of visual memory and reaction time were transformed with [ln(x + 1)] and [ln(x)], respectively, to achieve normal distribution.

The GWAS data utilized in our research originated from distinct cohorts or consortia, ensuring the absence of sample overlap.

### 2.3 Instrumental variable selection

Strictly quality control procedures were implemented to guarantee the robustness and precision of the causality among insulin resistance, brain cortical structure, and cognition. (1) SNPs strongly linked to insulin resistance phenotype (*p* < 5×10^−8^) were selected as IVs. Nevertheless, for SA and TH, the locus-wide significance level threshold was set to a relatively relaxed 1×10^−6^ to retain more IVs; (2) clumping procedure: removing IVs in linkage disequilibrium with r^2^<0.001, and clumping window=10000kb; (3) the minor allele frequency (MAF) > 0.01; (4) the F-statistic > 10, with the detailed calculation formula provided elsewhere (17); (5) harmonizing procedure: excluding palindromic and inconsistent IVs; (6) steiger filtering: the IVs were determined to be more predictive of exposure than outcome; (7) PhenoScanner V2 scanning: discarding the IVs correlated (*p* < 1×10^−5^) with confounding factors (23).

### 2.4 Statistical analysis

All analyses were conducted in the R version 4.1.2 environment using “TwoSampleMR” and “MRPRESSO” packages. The figures were drawn through FreeSurfer (version 7.2.0, https://surfer.nmr.mgh.harvard.edu) and Figdraw (https://www.figdraw.com).

#### 2.4.1 Primary analysis

Five complementary MR approaches with accommodated assumptions were conducted, including inverse variance weighted (IVW) (primary), MR Egger, weighted median, weighted mode, and simple mode. (1) The IVW is the optimal statistical approach assuming the validity of all IVs (24). However, the precision of IVW is susceptible to directional pleiotropy. (2) The MR Egger is a less efficient analytical method capable of providing unbiased estimations even if all IVs are pleiotropic, but it is substantially influenced by outliers (25). (3) The weighted median method is applicable when there are <50% invalid IVs and is robust to outliers (24). (4) The weighted mode persists steady even though IVs are disqualified or violate the pleiotropy hypothesis (26). (5) The simple mode is an unweighted empirical density function mode with relatively low statistical efficiency (27). As for multiple comparison correction, the statistically significant threshold was set at 0.025 (0.05/2) for the MR analysis between insulin resistance and cognition, and 0.0015 (0.05/34) for the MR analysis between insulin resistance and brain cortical structure. P-values between 0.05 and the specified threshold were considered nominally significant.

#### 2.4.2 Mediation analysis

The two-step univariable mediation MR analysis was implemented to investigate whether brain cortical structure mediates the causal pathway from insulin resistance to cognition outcome. The total effect of insulin resistance on cognition (c) can be decomposed into two components: (1) the direct effects of insulin resistance on cognition (without mediators, c’) and (2) the indirect effects mediated by brain cortical structure (a×b, where a represents the influence of insulin resistance on brain cortical structure and b represents the influence of brain cortical structure on cognition) (28). The mediation percentage was calculated using the equation (a×b)/c. Subsequently, we applied the delta method to calculate 95% confidence intervals (CI).

#### 2.4.3 Sensitivity analysis

Several sensitivity analyses were carried out to validate the reliability of the identified causal relationship. The Cochran’s Q statistics of MR Egger and IVW approaches were conducted to determine latent heterogeneity. A p-value larger than 0.05 indicated the absence of heterogeneity. The MR Egger intercept and Mendelian Randomised Multi-Effects Residuals and Heteroscedasticity (MR-PRESSO) approaches were concurrently employed to determine the latent horizontal pleiotropy. The intercept of MR Egger was nearly zero, and the p-value was greater than 0.05, demonstrating no pleiotropy. The leave-one-out analysis investigated whether the removal of a single SNP substantially influenced the total effect.

#### 2.4.4 Reverse MR analysis

For causality found to be significant or nominally significant in the forward MR analysis, we carried out the reverse MR analysis to verify the bidirectional relationship in the pathway. The threshold for IVs strongly correlated to cognition traits was set at 5×10^−8^, and the other procedures were similar to the forward MR analysis.

## 3 Results

### 3.1 Causality of insulin resistance on cognition

**Figure 2:**
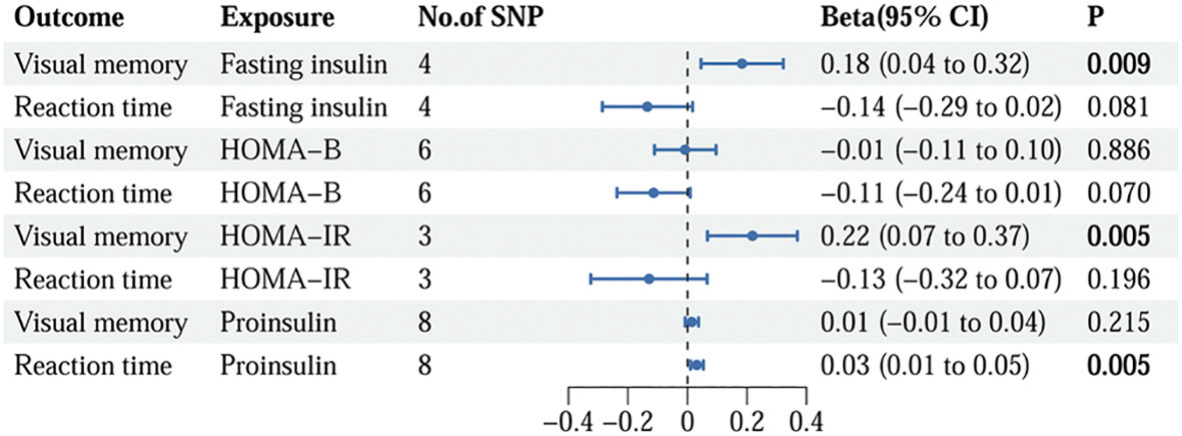
The causality of genetically predicted insulin resistance on cognition using IVW methods. IVW, inverse variance weighted; SNP, single nucleotide polymorphism; CI, confidence interval; HOMA-B, homeostasis model assessment beta-cell function; HOMA-IR, homeostasis model assessment insulin resistance.

Following the rigorous screening steps mentioned above, 9 SNPs with fasting insulin, 12 SNPs with HOMA-B, 8 SNPs with HOMA-IR, and 8 SNPs with proinsulin were selected as IVs, respectively. The comprehensive information for IVs of insulin resistance is listed in **Supplementary Table S4**. The IVs strongly linked to fasting insulin substantially overlapped with those in HOMA-IR. The relationships between insulin resistance phenotypes and cognition phenotypes are illustrated in **Figure 2**. The IVW method demonstrated that fasting insulin (beta=0.18, 95%CI=0.04 to 0.32, *p*=0.009) and HOMA-IR (beta=0.22, 95%CI=0.07 to 0.37, *p*=0.005) were causally correlated with visual memory. Additionally, a significant detrimental effect of proinsulin on reaction time was discovered using the IVW method (beta=0.03, 95%CI=0.01 to 0.05, *p*=0.005). However, no association was observed for HOMA-B.

### 3.2 Causality of insulin resistance on brain cortical structure

**Figure 3:**
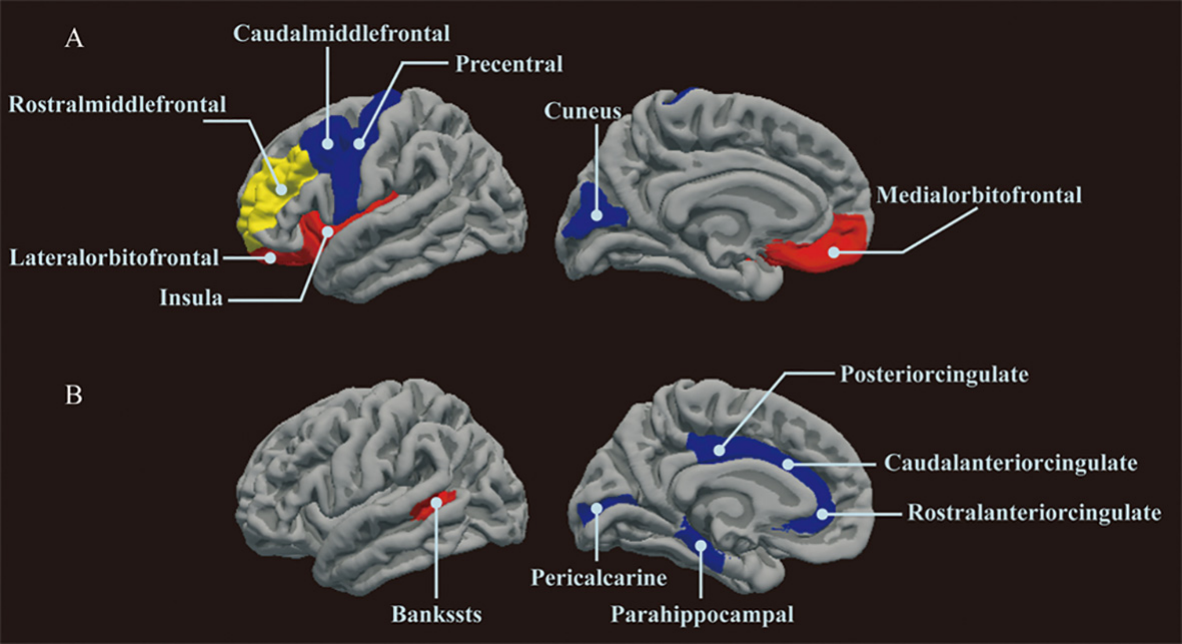
The results of MR analysis showed that insulin resistance potentially influenced the brain cortical structure of specific regions. **(A)** MR analysis results of insulin resistance on cortical surface area. **(B)** MR analysis results of insulin resistance on cortical thickness. Brain regions with positive and negative IVW-derived β values are shown in red and blue, respectively, brain region with negative IVW-derived β value and mediates the association between insulin resistance and cognition is shown in yellow. MR, Mendelian randomization; IVW, inverse variance weighting.

**Figure 4:**
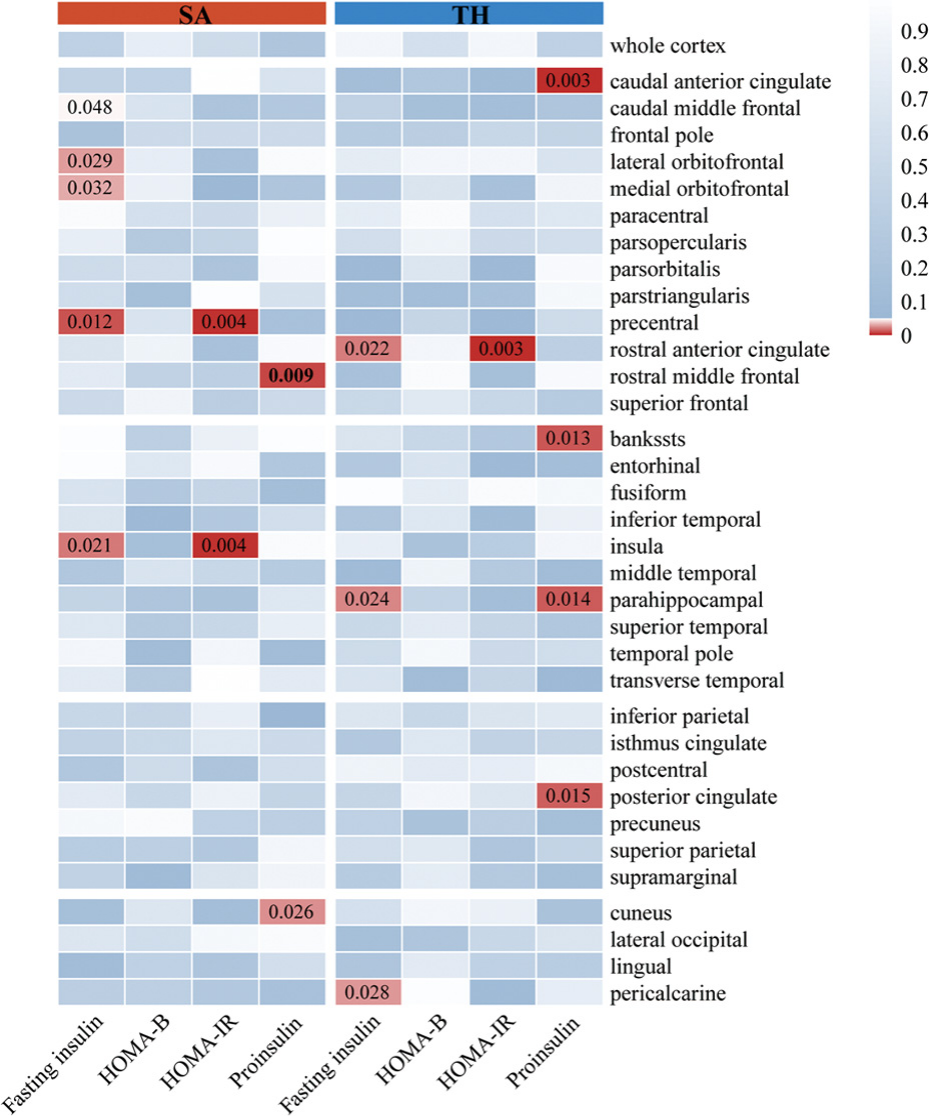
The results of MR analysis showed that insulin resistance potentially influenced the brain cortical structure of specific regions. The color of each block described the IVW-derived P-values of each MR analysis. P-values of <0.05 were shown in red, and P-values of >0.05 were shown in blue. MR, Mendelian randomization; IVW, inverse variance weighted; SA, surficial area; TH, thickness; HOMA-B, homeostasis model assessment beta-cell function; HOMA-IR, homeostasis model assessment insulin resistance.

As illustrated in **Figures 3**, **4**, the influence of insulin resistance on brain cortical structure, both protective and adverse, were determined. No significant causality was discovered for altering global SA and TH with insulin resistance. Concerning SA of specific regions, a higher level of proinsulin was nominally associated with a decreased SA of the rostral middle frontal (IVW: beta=-49.28, 95%CI=-86.30 to -12.27, *p*=0.009). The causal effects of HOMA-IR on SA of both precentral (IVW: beta=-161.91, 95%CI=-272.50 to -51.32, *p*=0.004) and insula (IVW: beta=84.15, 95%CI=26.30 to 142.00, *p*=0.004) turned borderline significant after Bonferroni adjustment. The fasting insulin and HOMA-IR determined both adverse impacts on the SA of the precentral and protective effects on the SA of the insula simultaneously. Respecting TH of specific regions, genetically predicted HOMA-IR was inversely related to TH of rostral anterior cingulate (IVW: beta=-0.09, 95%CI=-0.15 to -0.03, *p*=0.003). The proinsulin susceptibility was negatively linked to TH of the caudal anterior cingulate (IVW: beta=-0.03, 95%CI=-0.04 to -0.01, *p*=0.003). Nevertheless, limited evidence was noticed for the causality of HOMA-B on SA and TH. The detailed causality between each insulin resistance phenotype and brain cortical structure is presented in **Supplementary Tables S5**, **S6**.

### 3.3 Causality of brain cortical structure on cognition

Building upon the established causality between insulin resistance and brain cortical structure of specific regions. The SA and TH of specific regions were chosen as candidate mediators. Subsequently, we performed MR analysis on SA and TH of specific regions concerning cognition phenotypes. We observed that genetically determined SA of cuneus had a positive causal direction with reaction time (IVW: beta=2.79×10^-4^, 95%CI=5.99×10^-5^ to 4.98×10^-4^, *p*=0.013). The SA of the rostral middle frontal exhibited protective effects against longer reaction time (beta=-1.32×10^-4^, 95%CI=-2.04×10^-4^ to -5.91×10^-5^, *p*=0.0004), as indicated by robust IVW estimation. Consistent directional results were observed across all MR estimations.

### 3.4 Cortical structure mediates the causality of insulin resistance on cognition

**Figure 5:**
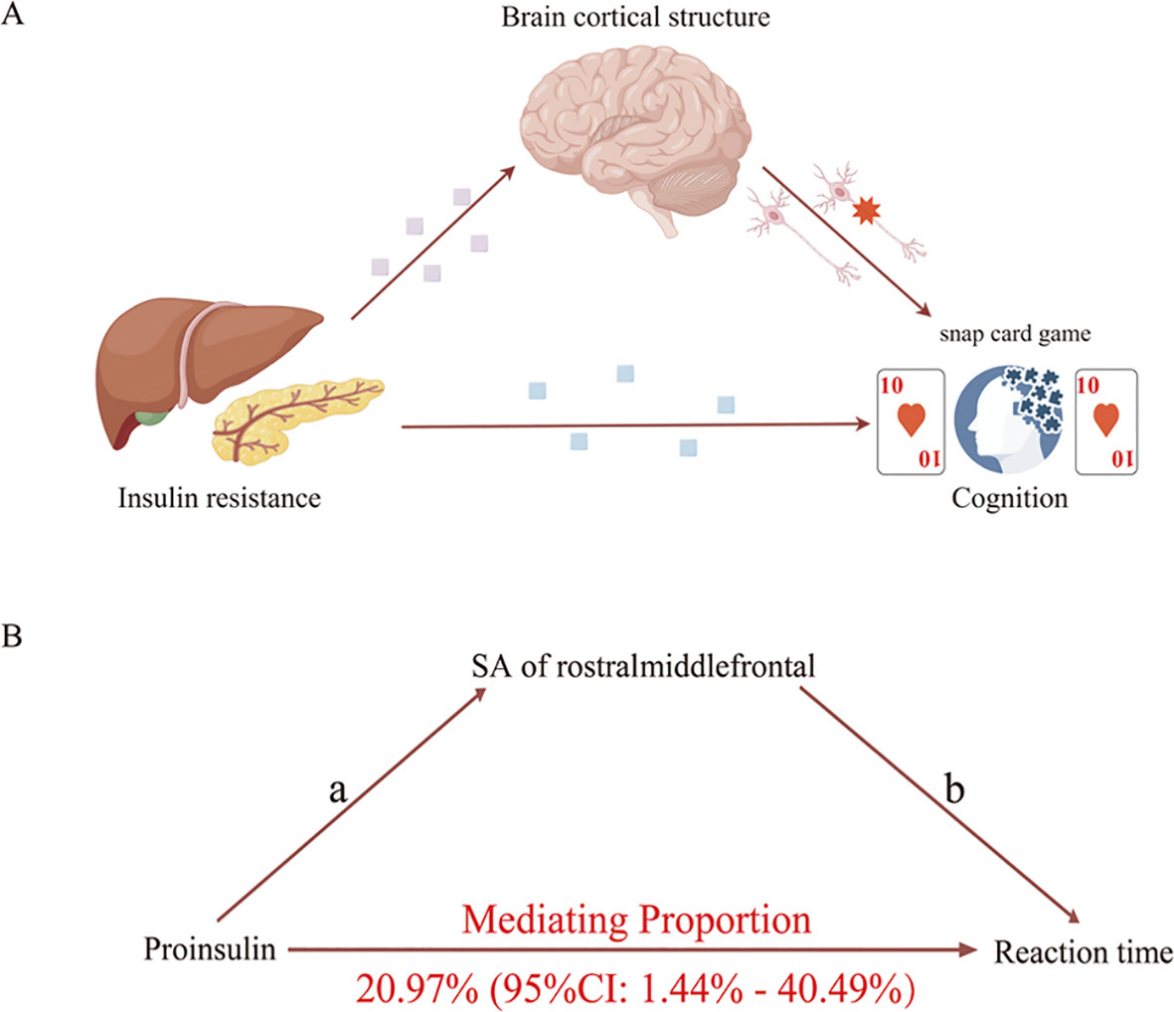
Schematic diagram of the mediation model. **(A)** Schematic diagram of the brain cortical structure’s effect on the pathway from insulin resistance to cognition. **(B)** Schematic diagram of the rostral middle frontal surficial area’s effect on the pathway from proinsulin to reaction time.

We analyzed the rostral middle frontal and cuneus’s SA as candidate mediators of the pathway from proinsulin to reaction time. Our study indicated that a higher level of proinsulin might result in lower SA of the rostral middle frontal, which in turn was related to a longer reaction time. However, the mediation model was invalid using the SA of cuneus as a mediator. As shown in **Figure 5**, the SA of the rostral middle frontal partially mediated the pathway from proinsulin to reaction time, accounting for 20.97% (95%CI=1.44% to 40.49%, *p*<0.05).

### 3.5 Sensitivity analysis

Estimation for Cochran’s Q statistic MR Egger and IVW tests indicated no significant heterogeneity in the causality. The MR-PRESSO global test showed a considerable *p* value and the MR-Egger intercept was nearly zero, emphasizing no significant horizontal pleiotropy (**Supplementary Table S7**). None underlying outliers were confirmed in the MR-PRESSO analysis. Furthermore, the observed causal estimate was not substantially affected by any strong driven SNP, as indicated by the leave-one-out test. The MR steiger filtering was determined to be more predictive of exposure than the outcome. Consequently, there was sufficient evidence supporting the robustness of our uncovering.

### 3.6 Reverse MR analysis

We further employed reverse MR analysis to evaluate the existence of bidirectional causality in the identified results from the forward analysis. We included up to 23 SNPs for visual memory and 58 for reaction time. Comprehensive information on the IVs is displayed in **Supplementary Table S8**. Results in **Supplementary Table S9** indicated no significant causality for genetically predicted reaction time on proinsulin, reaction time on SA of rostral middle frontal, and SA of rostral middle frontal on proinsulin. No evidence of heterogeneity and pleiotropy was found in the reverse MR analysis (**Supplementary Tables S10**).

## 4 Discussion

Through MR analysis, we investigated the causal influence of insulin resistance-related traits on cognition and evaluated the mediating effects of brain cortical structure in the pathway. Specifically, we identified that an elevated level of proinsulin, a marker of insulin resistance, led to increased reaction time, with the SA of rostral middle frontal mediated 20.97% of this effect. This study added suggestive evidence that brain cortical structure was crucial in the pathogenesis linking insulin resistance to the advancement of cognitive impairment.

Insulin resistance, a complicated phenotype, is typically assessed through various proxy indexes, with the euglycemic hyperinsulinemic glucose clamp technique considered the gold standard. Owing to the deficiency of updated large-scale GWAS on this gold standard measurement, we utilized four commonly employed surrogate markers in our MR analysis (29). Our study demonstrated a significant detrimental effect of insulin resistance traits on cognitive performance, specifically fasting insulin, HOMA-IR, and proinsulin, with no such effect observed for HOMA-B. It has been reported that compared to HOMA-B, higher HOMA-IR presented a closer connection with incident T2DM in Chinese adults (30). Given that diabetes is a well-established risk factor for cognitive impairment, this discrepancy could explain the lack of effect observed for HOMA-B. Furthermore, HOMA-IR, rather than HOMA-B, revealed a significant elevation in AD and a strong correlation with T-tau and P-tau in Cerebrospinal fluid (31). Collectively, these studies suggest that HOMA-IR is a more valuable indicator than HOMA-B. Considering the substantial overlap in IVs between fasting insulin and HOMA-IR, it is plausible that both are causally correlated with visual memory.

The adverse effect of higher insulin resistance on cognition aligned with several cross-sectional (32) and longitudinal studies (33). Contrary to the results mentioned above, one previous study conducted by Thankappan S et al. reported a null relationship between insulin resistance and AD with a relatively lower sample size (10). Surprisingly, Hooshmand B (11) followed 269 adults without dementia for 7 years, discovering the linkage between HOMA-IR and cognition in longitudinal analysis instead of at baseline. These discrepancies may reflect limitations inherent in observational research, such as confounding factors, reverse causality, and selection bias. Evidence from MR studies also showed a potential causal link between insulin resistance (34) and related traits (obesity) (35) with cognition. However, controversial MR analyses simultaneously existed, indicating no causality between HOMA-IR and cognition after controlling socioeconomic position and educational attainment (36). Additionally, prior MR analyses, using two large-scale population samples to explore causal associations (37), revealed genetic evidence of an association of HOMA-IR with verbal intelligence in the Generation Scotland: Scottish Family Health sample, whereas this correlation was not validated in the UK Biobank sample. Consequently, the inconsistent results across MR studies may attributed mainly to heterogeneity in the selection of participants, cohorts, sample size, and different phenotypes of insulin resistance and cognition. Further replication through randomized controlled trials is warranted.

Our study uncovered the latent causal influence of insulin resistance on brain cortical SA and TH. Post-mortem human brain studies have established the presence of insulin receptors in the brain, especially in the cortical regions (14). Consistent with our findings, the Rhineland Study, encompassing 973 participants, reported a similar inverse association between insulin resistance and the structure of the precentral cortex, temporal cortex, and cuneus (12). Our findings suggested that the specific brain cortical regions susceptible to insulin resistance are mainly distributed in the frontal, temporal, and limbic lobes. The underlying mechanisms for insulin resistance affecting brain cortical structure may be as follows. First, studies have shown that the increased cerebrospinal fluid Aβ42 (38), t-tau, and p-tau levels (31) were related to insulin resistance, which are pathological hallmarks of cognitive impairment disorder. Second, brain cortical glucose metabolism might be impacted by insulin resistance, which reflects the activity of neuronal and synaptic (16). Finally, insulin resistance may induce atherosclerosis, vascular endothelial dysfunction, oxidative stress, and chronic inflammation (39), contributing to cortical thinning and subsequent clinical events, including cognitive impairment. However, specific mechanisms remain unclear, necessitating further research in the future. Notably, the protective effects of genetically determined insulin resistance on the structure of orbitofrontal, insula, and bankssts are varied from logical expectation. Increased cortical SA or TH was generally considered a protective indicator against cognitive impairment. One plausible explanation is that compensatory hypertrophy or neural adaptation mitigates the adverse influence of higher insulin resistance on brain functional regions. Altogether, our study emphasizes the intricate and heterogeneous essence of insulin pathways within the brain.

Our research provided suggestive evidence that the SA of the rostral middle frontal mediates the effect of proinsulin on reaction time. It has been indicated that the structure of the rostral middle frontal was vulnerable in patients with T2DM, and the altered structure of this brain region held high diagnostic value for T2DM patients with mild cognitive impairment (40). The rostral middle frontal is a crucial component of the dorsolateral prefrontal cortex, playing a vital role in executive function. Additionally, the rostral middle frontal, along with the parietal lobe, constitutes a segment the dorsal attentional network (41). We employed the symbol matching test to evaluate reaction time, serving as an indicator of attention and executive function. However, another MR estimation did not support the causality among glycemia, brain structure and cognition (42). This study utilized T2DM and glycosylated hemoglobin as exposure, with hippocampal and white matter hyperintensity volumes as brain structural outcomes, which is largely different from ours. Consequently, we deduce that insulin resistance, rather than T2DM, exerts a direct influence on the brain structure. The SA of the rostral middle frontal may represent a latent pathophysiological process in the correlation between insulin resistance and cognition.

In the current survey, we primarily target the possible mediating role of phenotypes related to brain cortical structure, with approximately 80% of the mediation influence on cognition yet to be elucidated. The multi-model neuroimaging methods offer opportunities to unravel insulin resistance-related cognitive impairment (43). Unexplored mediating pathways may involve the macrostructures and microstructures, metabolism, perfusion, neural function, and brain network. Given that previous studies have established the causal effect of obesity (44) and blood lipids (45) on brain cortical structure, it is possible that these are essential candidate mediators as well. Future research is warranted to identify additional mediation factors along the pathway from insulin resistance to cognition.

This MR analysis exhibits multiple strengths. Firstly, the advantages of the MR statistic framework enable causality inference comparable to randomized controlled trials. Secondly, we incorporated comprehensive phenotypes related to insulin resistance, enhancing the integrity and rigor of the estimation. Thirdly, UK Biobank samples were excluded from the MR analysis of brain cortical structure. Thus, there was no sample overlap with the GWAS data used in our research, significantly reducing potential bias (46). Fourthly, sensitivity analyses and Bonferroni corrections were conducted sequentially to check the credibility of the discovered causality. Lastly, we implemented rigorous screening steps for mediators to guarantee the reliability and rationality of the mediation model. Nevertheless, certain limitations need to be considered. Firstly, the temporary measurement of insulin resistance is disposed to change over time without lifelong representation. Secondly, despite the absence of heterogeneity and pleiotropy in our findings, we cannot eliminate all potential biases and confounders. Thirdly, our research was restricted to individuals of European and American ancestry, minimizing population admixture confounding while limiting generalizability to other ethnic groups. Finally, the driven causality of insulin resistance on brain cortical structure did not withstand the Bonferroni correction, which just indicated suggestive causality. Caution explanations with additional validation in distinct cohorts are warranted.

Our research provided conceivable genetic evidence that elevated level of insulin resistance increased the susceptibility to cognitive impairment, with a partial mediation effect observed through the SA of the rostral middle frontal. Broader efforts are necessary to probe additional mediators. Our findings promote a better recognizing of the biological mechanisms underlying cognitive impairment induced by insulin resistance. Interventions to improve insulin sensitivity may serve as precautions against brain cortical atrophy and subsequent cognitive impairment. Nevertheless, further confirmation through randomized controlled trials is necessary.

## Data Availability

The original contributions presented in the study are included in the article/**Supplementary Material**. Further inquiries can be directed to the corresponding authors.
