# Supplementary material for: Genetically predicted brain cortical structure mediates the causality between insulin resistance and cognitive impairment
Source: Front Endocrinol (Lausanne). 2025 Jan 15;15:1443301. doi: 10.3389/fendo.2024.1443301 (PMC11774689; doi:10.3389/fendo.2024.1443301)
Supplement: Supplementary file 1 [file DataSheet1.pdf]

Supplementary Table S1. STROBE-MR Checklist. (Skrivankova et al., 2021)

Item No.	Section	Checklist item	Manuscript section and paragraph
**Title and abstract**			
1	Title and abstract	Indicate mendelian randomization (MR) as the study's design in the title and/or the abstract if that is a main purpose of the study.	**Abstract**
**Introduction**			
2	Background	Explain the scientific background and rationale for the reported study. What is the exposure? Is a potential causal relationship between exposure and outcome plausible? Justify why MR is a helpful method to address the study question.	**Introduction** paragraph 1-2
3	Objectives	State specific objectives clearly, including prespecified causal hypotheses (if any). State that MR is a method that, under specific assumptions, intends to estimate causal effects.	**Introduction** paragraph 3
**Methods**			
4	Study design and data sources	Present key elements of the study design early in the article. Consider including a table listing sources of data for all phases of the study. For each data source contributing to the analysis, describe the following:	
	a	Setting: Describe the study design and the underlying population, if possible. Describe the setting, locations, and relevant dates, including periods of recruitment, exposure, follow-up, and data collection, when available.	**Materials and methods** paragraph 1-5
	b	Participants: Report the eligibility criteria and the sources and methods of selection of participants. Report the sample size and whether any power or sample size calculations were carried out prior to the main analysis.	**Materials and methods** paragraph 1-5
	c	Describe measurement, quality control, and selection of genetic variants.	**Materials and methods** paragraph 1-5
	d	For each exposure, outcome, and other relevant variables, describe methods of assessment and diagnostic criteria for diseases.	**Materials and methods** paragraph 1-5
	e	Provide details of ethics committee approval and participant informed consent, if relevant.	Not relevant
5	Assumptions	Explicitly state the 3 core instrumental variable (IV) assumptions for the main analysis (relevance, independence, and exclusion restriction), as well assumptions for any additional or sensitivity analysis.	**Materials and methods** paragraph 5
6	Statistical methods: main analysis	Describe statistical methods and statistics used.	
	a	Describe how quantitative variables were handled in the analyses (ie, scale, units, model).	**Materials and methods** paragraph 7-8
	b	Describe how genetic variants were handled in the analyses and, if applicable, how their weights were selected.	**Materials and methods** paragraph 7-8
	c	Describe the MR estimator (eg, 2-stage least squares, Wald ratio) and related statistics. Detail the included covariates and, in case of 2-sample MR, whether the same covariate set was used for adjustment in the 2	**Materials and methods** paragraph 7-8
	d	Explain how missing data were addressed.	No missing data
	e	If applicable, indicate how multiple testing was addressed.	**Materials and methods** paragraph 7
7	Assessment of assumptions	Describe any methods or prior knowledge used to assess the assumptions or justify their validity.	**Materials and methods** paragraph 9-10
8	Sensitivity analyses and additional analyses	Describe any sensitivity analyses or additional analyses performed (eg, comparison of effect estimates from different approaches, independent replication, bias analytic techniques, validation of instruments, simulations).	**Materials and methods** paragraph 9-10
9	Software and preregistration		
	a	Name statistical software and package(s), including version and settings used.	**Materials and methods** paragraph 6
	b	State whether the study protocol and details were preregistered (as well as when and where).	No
**Results**			
10	Descriptive data		
	a	Report the numbers of individuals at each stage of included studies and reasons for exclusion. Consider use of a flow diagram.	**Materials and methods** paragraph 2-4
	b	Report summary statistics for phenotypic exposure(s), outcome(s), and other relevant variables (eg, means, SDs, proportions).	**Materials and methods** paragraph 2-4
	c	If the data sources include meta-analyses of previous studies, provide the assessments of heterogeneity across these studies.	No

	d	For 2-sample MR:<br>i. Provide justification of the similarity of the genetic variant–exposure associations between the exposure and outcome samples.<br>ii. Provide information on the number of individuals who overlap between the exposure and outcome studies.	**Materials and methods** paragraph 4
11	Main results		
	a	Report the associations between genetic variant and exposure and between genetic variant and outcome, preferably on an interpretable scale.	**Results** paragraph 1-4
	b	Report MR estimates of the relationship between exposure and outcome and the measures of uncertainty from the MR analysis, on an interpretable scale, such as odds ratio or relative risk per SD difference.	**Results** paragraph 1-4
	c	If relevant, consider translating estimates of relative risk into absolute risk for a meaningful time period.	**Results** paragraph 1-4
	d	Consider plots to visualize results (eg, forest plot, scatterplot of associations between genetic variants and outcome vs between genetic variants and exposure).	**Results** paragraph 1-4
12	Assessment of assumptions		
	a	Report the assessment of the validity of the assumptions.	**Results** paragraph 5
	b	Report any additional statistics (eg, assessments of heterogeneity across genetic variants, such as I2, Q statistic, or E-value).	**Results** paragraph 5
13	Sensitivity analyses and additional analyses		
	a	Report any sensitivity analyses to assess the robustness of the main results to violations of the assumptions.	**Results** paragraph 5
	b	Report results from other sensitivity analyses or additional analyses.	**Results** paragraph 5
	c	Report any assessment of the direction of the causal relationship (eg, bidirectional MR).	**Results** paragraph 6
	d	When relevant, report and compare with estimates from non-MR analyses.	No
	e	Consider additional plots to visualize results (eg, leave-one-out analyses).	**Results** paragraph 5
**Discussion**			
14	Key results	Summarize key results with reference to study objectives.	**Discussion** paragraph 1
15	Limitations	Discuss limitations of the study, taking into account the validity of the IV assumptions, other sources of potential bias, and imprecision. Discuss both direction and magnitude of any potential bias and any efforts to address	**Discussion** paragraph 7
16	Interpretation		
	a	Meaning: Give a cautious overall interpretation of results in the context of their limitations and in comparison with other studies.	**Discussion** paragraph 2-6
	b	Mechanism: Discuss underlying biological mechanisms that could drive a potential causal relationship between the investigated exposure and the outcome, and whether the gene-environment equivalence assumption is reasonable. Use causal language carefully, clarifying that IV estimates may provide causal effects only under	**Discussion** paragraph 2-6
	c	Clinical relevance: Discuss whether the results have clinical or public policy relevance, and to what extent they inform effect sizes of possible interventions.	**Discussion** paragraph 2-6
17	Generalizability	Discuss the generalizability of the study results (a) to other populations, (b) across other exposure periods/timings, and (c) across other levels of exposure.	**Discussion** paragraph 7
**Other Information**			
18	Funding	Describe sources of funding and the role of funders in the present study and, if applicable, sources of funding for the databases and original study or studies on which the present study is based.	**Funding**
19	Data and data sharing	Provide the data used to perform all analyses or report where and how the data can be accessed, and reference these sources in the article. Provide the statistical code needed to reproduce the results in the article or report whether the code is publicly accessible and, if so, where.	**Data Availability**
20	Conflicts of interest	All authors should declare all potential conflicts of interest.	**Conflict of Interest**

Reference:

Skrivankova, V.W., Richmond, R.C., Woolf, B.A.R., Yarmolinsky, J., Davies, N.M., Swanson, S.A., et al. (2021). Strengthening the Reporting of Observational Studies in Epidemiology Using Mendelian Randomization: The STROBE-MR Statement. JAMA 326, 1614-1621. doi: 10.1001/jama.2021.18236

Supplementary Table S2. Details of each insulin resistance cohort of European ancestry in the GWAS meta-analysis.

**Phenotype: Fasting insulin, HOMA-B, HOMA-IR**						
**Cohort**	**Geographical origin**	**Samples with fasting insulin phenotype: N all (%males / %female)**	**Samples with HOMA-B phenotype: N all (%males / %female)**	**Samples with HOMA-IR phenotype: N all (%males / %female)**	**age [Mean (sd) males / Mean (sd) females], years**	**Adjustments**
**CHS**	USA	1723 (43 / 57)	1724 (43 / 57)	1723 (43 / 57)	73.4 (5.7) / 73.1 (5.5)	Age, sex, study site
**FHS**	USA	6,040 (46.4 / 53.6)	6,038 (46.3 / 53.7)	6,040 (46.4 / 53.6)	45.9 (11.5) / 46.0 (11.6)	Sex-specific residuals adjusted for age and age^2
**DGI**	Finland, Sweden	951 (45.8 / 54.2)	936 (45.9 / 54.1)	936 (45.9 / 54.1)	58.3 (10.3) / 59.2 (10.0)	Age, sex, recruitment region
**BLSA**	USA	762 (55.3 / 44.8)	747 (55.8 / 44.2)	747 (55.8 / 44.2)	66.8 (16.8) / 62.9 (18.2)	Age, sex, and PC
**FUSION**	Finland	1,115 (49.2 / 50.8)	1,111 (49.1 / 50.9)	1,113 (49.1 / 50.9)	63.15 (7.61) / 63.52 (7.31)	Sex, age, age^2, study
**CoLaus**	Switzerland	4,457 (48 / 52)	4,303 (47 / 53)	4,311 (47 / 53)	52.26 (10.74) / 53.73 (10.73)	Age and sex
**InCHIANTI**	Italy	1,026 (44.83 / 55.17)	1,018 (44.70 / 55.30)	1,023 (44.67 / 55.33)	67.32 (15.16) / 69.24 (15.70)	Age and sex
**NFBC1966**	Finland	4,175 (48.55 / 51.45)	4,166 (48.6 / 51.4)	4,166 (48.6 / 51.4)	31 (-) / 31 (-)	Sex, 3 PCs
**NTR / NESDA**	Netherlands	1,229 (35.6 / 64.4)	1,196 (35.5 / 64.5)	1,196 (35.5 / 64.5)	46.0 (13.4) / 42.1 (13.1)	Age, sex, cohort
**Rotterdam Study**	Netherlands	2,781(41.6 / 58.4)	1,801 (42.4 / 57.6)	1,802 (42.4 / 57.6)	63.8 (5.5) / 64.2 (6.1)	Age and sex
**PROCARDIS**	UK, Sweden, Italy, Germany	4164 (76.3 / 23.7)	3075 (81.4 / 18.6)	3075 (81.4 / 18.6)	57.9 (8.4) / 61.0 (7.7)	Age and sex
**Sorbs**	Germany	673 (40.6 / 59.4)	673 (40.6 / 59.4)	672 (40.5 / 59.5)	47 (16.3) / 46.7 (15.9)	Age and sex
**ERF**	Netherlands	1063 (39.23 / 60.77)	1004 (39.94 / 60.06)	1079 (39.2 / 60.8)	49.77 (14.6) / 48.02 (14.42)	Age and sex
**CROAS (Vis Study)**	Croatia	657 (42.01 / 57.99)	655 (41.83 / 58.17)	657 (42.01 / 57.99)	54.74 (14.94) / 55.2 (15.75)	Age and sex
**ORCADES (Orkney)**	Scotland	662 (45.47 / 54.53)	662 (45.47 / 54.53)	663 (45.55 / 54.45)	54.03 (15.41) / 52.99 (15.41)	Age and sex
**AGES**	Iceland	2850(59.7 / 40.3)	2850(59.7 / 40.3)	2850(59.7 / 40.3)	76.65 (5.35) / 76.29 (5.59)	Age and sex
**ARIC**	USA	8372 (46.4 / 53.6)	8370 (46.4 / 53.6)	8372 (46.4 / 53.6)	54.5 (5.67) / 53.8 (5.65)	Age, sex, center
**BSN**	Australia	1174 (41.2 / 58.8)	1174 (41.2 / 58.8)	1174 (41.2 / 58.8)	51.99 (17.03) / 52.38 (17.12)	Age and sex
**FamHS**	US	552 (52.72 / 47.28)	550 (52.91 / 47.09)	552 (52.72 / 47.28)	50.69 (12.45) / 56.47 (10.40)	Age, sex, field center
**Fenland**	UK	1,398 (44 / 56)	1,392 (44 / 56)	1,392 (44 / 56)	44.50 (7.31) / 45.33 (7.18)	Age, sex (and BMI)
**French Adult Controls**	France	715 (25 / 75)	617 (24 / 76)	617 (24 / 76)	53.1 (5.59) / 49.52 (8.53)	Age, sex (and BMI)
**GENOA**	USA	1147 (44 / 56)	1147 (44 / 56)	1147 (44 / 56)	55.1 (11.1) / 54.4 (10.8)	Age, sex, BMI
**GenomEUtwin**	n, Denmark, Finland, The Neth	402 (0 / 100)	391 (0 / 100)	391 (0 / 100)	51.84 (21.21)	Age, cohort
**HABC**	United States	1559 (52.5 / 47.5)	1558 (52.6 / 47.4)	1558 (52.6 / 47.4)	73.9 (2.9) / 73.6 (2.8)	Age, Sex, (and BMI)
**Health2000**	Finland	1.923 (49.0 / 51.0)	1,923 (49.0 / 51.0)	1.923 (49.0 / 51.0)	49.2 (10.4) / 52.2 (11.5)	Age, age^2 and sex
**Korcula**	Croatia	758 (33.51 / 66.49)	758 (33.51 / 66.49)	758 (33.51 / 66.49)	55.85 (14.61) / 53.87 (13.13)	Age and sex

**Phenotype: Proinsulin**					
**Cohort**	**Geographical origin**	**N all (%males / %female)**	**Fasting intact proinsulin [Mean (sd) males / Mean (sd) females], pmol/l**	**Age [Mean (sd) males/Mean (sd) females], years**	**Adjustments**
**FHS**	USA	5759 (46.7 / 53.3)	12.29 (10.88) / 9.24 (7.50)	48.5 (13.4) / 48.9 (13.8)	Fasting insulin, age, and sex
**PROCARDIS**	Germany, Italy, Sweden, UK	3259 (73.8 / 26.2)	5.50 (6.78) / 5.11 (6.83)	58.3 (7.84) / 61.6 (7.09)	Fasting insulin, age, and sex
**Fenland**	UK	1372 (43.8 / 56.2)	4.18 (2.86) / 2.82 (2.00)	44.40 (7.28) / 45.37 (7.20)	Fasting insulin, age, and sex
**DGI**	Sweden, Finland	311 (51.5 / 48.5)	13.13 (8.92) / 10.25 (6.4)	58.85 (10.03) / 59.92	Fasting insulin, age, and sex

Notes: GWAS, genome-wide association studies; HOMA-B, homeostasis model assessment beta-cell function; HOMA-IR, homeostasis model assessment insulin resistance; sd, standard deviation; BMI, Body Mass Index.

Supplementary Table S3. Details of each brain cortical structure cohort of European ancestry in the GWAS meta-analysis.

**Phenotype: cortical surficial area and thickness of the brain**				
**Cohort**	**Total N**	**Females**	**Mean Age**	**SD Age**
1000BRAINS	775	346	67.3	6.7
ADNI1	735	299	74.8	6.8
ADNI2GO	649	297	72.4	7.1
ALSPAC	391	0	19.6	0.9
ASRB	233	138	38.5	11.3
BETULA	311	169	62.4	13.3
BIG-Affy	1180	688	22.6	3.8
BIG-PsychChip	432	206	22.5	4.4
BONN	102	0	38.2	6.6
BrainScale	242	131	10.0	1.3
CARDIFF	270	194	24.8	6.9
DNS-V3	324	168	19.7	1.2
DNS-V4	191	108	19.9	1.2
EPIGEN	178	104	38.4	13.2
FOR2107	785	474	34.4	13.0
GIG	283	168	24.2	2.4
GSP	442	251	21.4	3.2
HUBIN	177	55	41.9	8.2
HUNT	876	462	58.9	4.2
IMAGEN	1358	725	14.6	0.4
IMpACT	238	140	40.8	12.0
LBC1936	604	285	72.7	0.7
LIBD	484	214	33.2	10.1
MCIC	162	56	33.7	11.2
MooDS	282	129	33.6	9.8
MPIP	550	318	48.3	13.3
MPRC	387	205	37.2	14.6
MÜNSTER	985	561	35.8	12.1
NCNG	321	218	51.6	16.7

NESDA	254	171	37.5	10.2
NeuroIMAGE	210	68	17.1	3.2
NTR	322	197	29.4	11.0
OATS	360	237	70.5	5.1
PAFIP	112	42	28.3	8.0
PDNZ	164	56	68.2	7.8
PING	337	151	11.8	4.7
PPMI	414	137	61.7	9.6
QTIM	996	645	22.4	3.3
SHIP	1118	579	55.8	12.8
SHIP-Trend	891	499	50.4	13.5
Sydney MAS	494	274	78.4	4.7
SYS	1675	884	28.3	17.4
TCD-NUIG	192	108	29.9	10.4
TOP	505	253	35.2	10.2
TOP3T	400	181	33.2	11.9
UiO2016	229	98	31.8	10.0
UiO2017	308	173	42.1	17.9
UMCU	698	373	33.1	14.1

Notes: GWAS, genome-wide association studies; sd, standard deviation.

Supplementary Table S4. Detailed information of instrumental variables for insulin resistance used in MR analyses

Phenotype	SNP	Effect allele	Other allele	MAF	Beta	SE	P-value	F
Fasting insulin	rs10857297	A	G	0.408	0.017	0.0029	3.96E-09	34.364
Fasting insulin	rs12328675	T	C	0.147	0.029	0.0041	1.48E-12	50.030
Fasting insulin	rs1260326	T	C	0.420	-0.019	0.0027	6.20E-13	49.520
Fasting insulin	rs17036328	T	C	0.097	0.023	0.0038	2.97E-09	36.634
Fasting insulin	rs2114912	T	G	0.172	0.024	0.0036	3.36E-11	44.444
Fasting insulin	rs2126259	T	C	0.088	0.023	0.0041	1.53E-08	31.469
Fasting insulin	rs2785990	T	C	0.314	0.016	0.0028	2.72E-08	32.653
Fasting insulin	rs2972144	A	G	0.367	-0.021	0.0028	4.50E-14	56.250
Fasting insulin	rs4646949	T	G	0.274	0.018	0.0031	8.40E-09	33.715
HOMA-B	rs10258074	A	T	0.483	0.017	0.0026	1.58E-11	42.751
HOMA-B	rs10501320	C	G	0.292	0.017	0.0030	7.17E-09	32.111
HOMA-B	rs10830963	C	G	0.300	0.033	0.0031	4.23E-26	113.319
HOMA-B	rs11558471	A	G	0.252	-0.016	0.0027	1.94E-09	35.117
HOMA-B	rs13273088	A	G	0.195	-0.022	0.0035	3.92E-10	39.510
HOMA-B	rs174556	T	C	0.296	0.016	0.0027	2.08E-09	35.117
HOMA-B	rs2943640	A	C	0.372	-0.016	0.0026	3.88E-10	37.870
HOMA-B	rs35747	A	G	0.125	0.019	0.0032	1.90E-09	35.254
HOMA-B	rs3757840	T	G	0.491	-0.018	0.0026	3.16E-12	47.929
HOMA-B	rs4237150	C	G	0.456	-0.015	0.0025	2.42E-09	36.000
HOMA-B	rs557462	T	C	0.372	-0.036	0.0026	2.45E-42	191.716
HOMA-B	rs7903146	T	C	0.279	-0.019	0.0029	4.26E-11	42.925
HOMA-IR	rs12328675	T	C	0.147	0.029	0.0044	1.87E-11	43.440
HOMA-IR	rs1260326	T	C	0.420	-0.024	0.0029	6.67E-17	68.490
HOMA-IR	rs17036328	T	C	0.097	0.025	0.0041	1.05E-09	37.180
HOMA-IR	rs2820237	A	G	0.310	0.020	0.0035	1.94E-08	32.653
HOMA-IR	rs2972144	A	G	0.367	-0.021	0.0029	1.83E-12	52.438
HOMA-IR	rs35755	A	G	0.158	-0.024	0.0036	2.17E-11	44.444
HOMA-IR	rs4240624	A	G	0.075	-0.025	0.0045	3.13E-08	30.864
HOMA-IR	rs4691380	T	C	0.345	-0.018	0.0031	8.31E-09	33.715
Proinsulin	rs10501320	C	G	0.292	-0.110	0.0080	2.05E-45	189.063

Proinsulin	rs11558471	A	G	0.252	0.057	0.0079	4.21E-13	52.059
Proinsulin	rs11603334	A	G	0.129	0.130	0.0100	3.25E-38	169.000
Proinsulin	rs4502156	T	C	0.420	0.048	0.0072	3.81E-11	44.444
Proinsulin	rs4790333	T	C	0.398	0.040	0.0071	2.16E-08	31.740
Proinsulin	rs6101962	A	G	0.107	0.150	0.0220	9.89E-12	46.488
Proinsulin	rs6235	C	G	0.267	-0.058	0.0078	7.71E-14	55.293
Proinsulin	rs7903146	T	C	0.279	0.067	0.0077	3.48E-18	75.713

Notes: MR, Mendelian randomization; SNP, single nucleotide polymorphisms; MAF, minor allele frequency; Beta, estimate coefficient; SE, standard error of coefficient estimate; HOMA-B, homeostasis model assessment beta-cell function; HOMA-IR, homeostasis model assessment insulin resistance.

Supplementary Table S5. MR analysis results of insulin resistance on cortical surface area.

Causal effects were estimated using five two-sample MR methods (IVW, MR Egger, weighted median, weighted mode, and simple mode). P value < 0.05 was regarded as nominally significant. P value < 0.0015 was considered significant.

Exposure	Outcome	Lobe	IVs N	IVW					MR Egger					Weighted Median					Weighted Mode					Simple Mode				
				Estimate (β)	SE	*P* value	95% lower CI	95% upper CI	Estimate (β)	SE	*P* value	95% lower CI	95% upper CI	Estimate (β)	SE	*P* value	95% lower CI	95% upper CI	Estimate (β)	SE	*P* value	95% lower CI	95% upper CI	Estimate (β)	SE	*P* value	95% lower CI	95% upper CI
Fasting insulin	whole cortex	global	9	-2150.776	2523.978	0.394	-7097.774	2796.222	-9530.295	14425.713	0.530	-37804.692	18744.102	-451.053	3337.607	0.892	-6992.762	6090.656	846.442	4607.057	0.859	-8183.389	9876.274	467.414	5113.246	0.929	-9554.548	10489.376
Fasting insulin	caudal anterior cingulate	frontal	9	-15.867	19.502	0.416	-54.091	22.356	-87.683	115.883	0.474	-314.813	139.448	-20.162	24.239	0.406	-67.669	27.346	24.186	49.571	0.639	-72.973	121.345	-59.401	45.906	0.232	-149.376	30.575
Fasting insulin	caudal middle frontal	frontal	9	-92.546	46.792	0.048	-184.257	-0.834	-176.927	267.384	0.529	-701.000	347.147	-63.178	61.300	0.303	-183.327	56.970	-61.625	80.600	0.466	-219.601	96.351	-61.625	88.742	0.507	-235.559	112.310
Fasting insulin	frontal pole	frontal	9	-6.828	5.388	0.205	-17.389	3.734	-32.676	30.776	0.324	-92.997	27.644	-3.191	6.969	0.647	-16.850	10.468	-1.162	10.730	0.916	-22.193	19.869	-1.322	10.722	0.905	-22.338	19.693
Fasting insulin	lateral orbitofrontal	frontal	9	72.688	33.277	0.029	7.464	137.911	65.328	199.997	0.753	-326.667	457.323	49.569	44.886	0.269	-38.408	137.546	27.474	65.556	0.686	-101.015	155.963	53.816	73.317	0.484	-89.885	197.517
Fasting insulin	medial orbitofrontal	frontal	9	53.101	24.728	0.032	4.633	101.569	-121.568	141.287	0.418	-398.490	155.355	54.108	32.669	0.098	-9.923	118.138	53.250	56.320	0.372	-57.137	163.636	52.396	56.078	0.377	-57.516	162.309
Fasting insulin	paracentral	frontal	9	-2.290	45.448	0.960	-91.367	86.788	-131.227	273.080	0.645	-666.463	404.010	11.121	38.081	0.770	-63.517	85.760	21.406	55.174	0.708	-86.735	129.548	21.406	72.645	0.776	-120.978	163.791
Fasting insulin	parsopercularis	frontal	9	-10.784	41.083	0.793	-91.306	69.738	-318.948	221.477	0.193	-753.043	115.147	-48.845	45.060	0.278	-137.163	39.474	-74.068	69.333	0.317	-209.961	61.825	-83.250	72.640	0.285	-225.624	59.123
Fasting insulin	parsorbitalis	frontal	9	-7.086	11.861	0.550	-30.333	16.162	-18.445	72.297	0.806	-160.146	123.257	-12.015	14.728	0.415	-40.882	16.851	-32.827	24.320	0.214	-80.494	14.839	24.583	26.056	0.373	-26.487	75.652
Fasting insulin	parstriangularis	frontal	9	29.523	50.990	0.563	-70.418	129.463	-384.854	267.822	0.194	-909.786	140.078	-9.939	44.150	0.822	-96.474	76.596	-17.315	65.980	0.800	-146.636	112.007	-17.315	67.387	0.804	-149.393	114.764
Fasting insulin	precentral	frontal	9	-145.684	58.179	0.012	-259.714	-31.654	123.043	332.945	0.723	-529.529	775.616	-117.057	74.284	0.115	-262.654	28.540	-79.851	124.380	0.539	-323.637	163.935	-88.141	108.557	0.440	-300.913	124.632
Fasting insulin	rostral anterior cingulate	frontal	9	9.462	21.190	0.655	-32.071	50.995	22.783	129.221	0.865	-230.491	276.057	18.393	25.022	0.462	-30.650	67.436	32.870	34.781	0.372	-35.300	101.041	32.069	37.216	0.414	-40.875	105.013
Fasting insulin	rostral middle frontal	frontal	9	-32.575	100.486	0.746	-229.529	164.378	569.078	569.362	0.351	-546.873	1685.028	57.683	100.079	0.564	-138.473	253.838	94.151	143.794	0.531	-187.686	375.988	94.151	152.465	0.554	-204.681	392.983
Fasting insulin	superior frontal	frontal	9	55.682	88.102	0.527	-116.998	228.361	983.267	404.212	0.045	191.012	1775.521	-21.641	100.520	0.830	-218.660	175.377	-139.895	217.463	0.538	-566.123	286.333	-130.744	191.698	0.514	-506.471	244.983
Fasting insulin	bankssts	temporal	9	0.407	29.535	0.989	-57.482	58.297	30.199	180.103	0.872	-322.802	383.201	11.261	27.874	0.686	-43.373	65.894	34.860	52.333	0.524	-67.712	137.433	-59.614	61.144	0.358	-179.457	60.228
Fasting insulin	entorhinal	temporal	9	0.255	11.607	0.982	-22.494	23.005	-35.297	66.417	0.612	-165.475	94.881	-5.617	15.298	0.713	-35.602	24.367	-11.377	27.655	0.692	-65.581	42.827	-11.001	26.078	0.684	-62.113	40.112
Fasting insulin	fusiform	temporal	9	-28.569	60.751	0.638	-147.641	90.504	-322.887	353.610	0.392	-1015.962	370.188	-68.708	64.100	0.284	-194.344	56.928	-143.835	95.436	0.170	-330.889	43.220	-138.136	96.166	0.189	-326.622	50.349
Fasting insulin	inferior temporal	temporal	9	-28.157	62.591	0.653	-150.835	94.521	-535.653	328.915	0.147	-1180.327	109.021	-14.270	71.545	0.842	-154.497	125.958	-34.357	110.197	0.763	-250.344	181.630	14.314	125.048	0.912	-230.779	259.408
Fasting insulin	insula	temporal	9	94.534	40.812	0.021	14.542	174.526	-217.373	218.492	0.353	-645.618	210.871	90.745	45.424	0.046	1.714	179.777	113.536	71.400	0.150	-26.408	253.480	132.325	68.633	0.090	-2.195	266.846
Fasting insulin	middle temporal	temporal	9	47.879	43.206	0.268	-36.805	132.563	247.506	246.981	0.350	-236.578	731.589	27.200	57.772	0.638	-86.034	140.434	9.471	91.523	0.920	-169.914	188.856	-0.007	83.896	1.000	-164.443	164.428
Fasting insulin	parahippocampal	temporal	9	-14.947	19.140	0.435	-52.461	22.566	-20.874	116.758	0.863	-249.720	207.972	-6.262	19.922	0.753	-45.310	32.785	10.321	33.895	0.769	-56.114	76.756	11.820	35.842	0.750	-58.429	82.070
Fasting insulin	superior temporal	temporal	9	23.106	58.310	0.692	-91.181	137.393	363.311	331.245	0.309	-285.929	1012.551	55.795	55.265	0.313	-52.524	164.115	101.004	80.836	0.247	-57.434	259.442	84.016	94.482	0.400	-101.168	269.200
Fasting insulin	temporal pole	temporal	9	1.288	10.188	0.899	-18.680	21.256	-115.821	49.363	0.051	-212.573	-19.070	8.857	11.842	0.454	-14.352	32.067	11.779	18.537	0.543	-24.552	48.111	11.436	17.137	0.523	-22.152	45.025
Fasting insulin	transverse temporal	temporal	9	-3.240	9.971	0.745	-22.784	16.304	-50.822	58.121	0.411	-164.738	63.094	1.364	11.567	0.906	-21.309	24.036	2.763	16.232	0.869	-29.052	34.578	3.111	17.091	0.860	-30.388	36.610
Fasting insulin	inferior parietal	parietal	9	77.037	107.316	0.473	-133.302	287.376	-363.661	633.686	0.584	-1605.687	878.364	92.461	113.555	0.416	-130.108	315.029	279.775	205.173	0.210	-122.364	681.914	292.134	201.568	0.185	-102.940	687.208
Fasting insulin	isthmus cingulate	parietal	9	19.347	23.999	0.420	-27.690	66.385	-210.219	117.158	0.116	-439.850	19.411	-0.979	27.585	0.972	-55.045	53.088	-8.666	37.043	0.821	-81.270	63.939	-3.400	42.230	0.938	-86.171	79.370
Fasting insulin	postcentral	parietal	9	-54.870	48.933	0.262	-150.779	41.039	-46.526	279.986	0.873	-595.299	502.247	-60.726	61.420	0.323	-181.110	59.658	-55.257	102.674	0.605	-256.498	145.984	-57.137	104.710	0.600	-262.369	148.095
Fasting insulin	posterior cingulate	parietal	9	6.408	19.736	0.745	-32.275	45.091	-91.684	112.802	0.443	-312.775	129.407	7.655	26.190	0.770	-43.677	58.988	6.267	37.333	0.871	-66.906	79.440	6.722	36.085	0.857	-64.005	77.448
Fasting insulin	precuneus	parietal	9	5.229	53.287	0.922	-99.213	109.671	-225.412	313.557	0.496	-839.983	389.160	12.892	67.991	0.850	-120.370	146.154	17.651	96.452	0.859	-171.395	206.696	79.510	117.655	0.518	-151.093	310.114
Fasting insulin	superior parietal	parietal	9	-69.049	71.716	0.336	-209.613	71.515	122.587	410.279	0.774	-681.559	926.733	-44.236	86.226	0.608	-213.240	124.768	-17.945	136.742	0.899	-285.958	250.069	-23.744	125.001	0.854	-268.746	221.257
Fasting insulin	supramarginal	parietal	9	46.116	58.137	0.428	-67.833	160.065	-517.181	332.328	0.164	-1168.543	134.182	98.996	77.616	0.202	-53.132	251.124	106.490	121.795	0.407	-132.227	345.207	112.727	126.588	0.399	-135.385	360.839
Fasting insulin	cuneus	occipital	9	-42.819	31.030	0.168	-103.637	17.999	-33.634	189.614	0.864	-405.277	338.009	-20.416	35.878	0.569	-90.736	49.904	11.328	49.568	0.825	-85.826	108.482	6.058	51.475	0.909	-94.833	106.950
Fasting insulin	lateral occipital	occipital	9	27.097	66.512	0.684	-103.267	157.460	-606.212	380.161	0.155	-1351.328	138.904	7.413	88.245	0.933	-165.548	180.374	-40.292	134.095	0.771	-303.117	222.534	93.428	142.951	0.532	-186.756	373.613
Fasting insulin	lingual	occipital	9	-89.498	57.998	0.123	-203.173	24.177	235.337	331.534	0.501	-414.469	885.143	-88.254	68.714	0.199	-222.933	46.425	-77.580	96.394	0.444	-266.513	111.353	-84.314	100.827	0.427	-281.934	113.306
Fasting insulin	pericalcarine	occipital	9	-40.879	44.634	0.360	-128.361	46.602	-117.759	270.959	0.677	-648.838	413.321	-24.336	48.604	0.617	-119.599	70.928	0.431	72.542	0.995	-141.750	142.613	13.185	74.006	0.863	-131.867	158.236
HOMA-B	whole cortex	global	10	-664.461	2484.805	0.789	-5534.679	4205.757	-8250.089	7799.987	0.321	-23538.064	7037.886	-2314.089	2867.702	0.420	-7934.785	3306.607	-3028.605	3132.922	0.359	-9169.132	3111.922	-2720.678	4404.173	0.552	-11352.858	5911.502
HOMA-B	caudal anterior cingulate	frontal	10	-14.784	17.428	0.396	-48.943	19.375	-8.610	58.127	0.886	-122.539	105.319	-28.330	19.774	0.152	-67.088	10.428	-27.382	21.212	0.229	-68.957	14.193	-33.852	30.743	0.299	-94.107	26.404
HOMA-B	caudal middle frontal	frontal	10	-19.891	42.082	0.636	-102.372	62.590	-2.284	140.339	0.987	-277.350	272.781	-17.820	51.592	0.730	-118.941	83.301	-15.120	60.336	0.808	-133.379	103.140	-33.417	86.308	0.708	-202.581	135.748
HOMA-B	frontal pole	frontal	10	-2.869	4.433	0.518	-11.557	5.820	-11.079	13.950	0.450	-38.422	16.263	-4.814	5.968	0.420	-16.511	6.884	7.356	8.288	0.398	-8.888	23.600	6.800	10.925	0.549	-14.612	28.212
HOMA-B	lateral orbitofrontal	frontal	10	7.724	27.408	0.778	-45.995	61.443	33.036	86.247	0.712	-136.008	202.080	14.917	33.890	0.660	-51.507	81.341	14.425	42.917	0.744	-69.692	98.541	4.386	52.583	0.935	-98.677	107.448
HOMA-B	medial orbitofrontal	frontal	10	-4.836	20.354	0.812	-44.729	35.057	-49.434	64.052	0.462	-174.976	76.107	-23.730	26.717	0.374	-76.095	28.635	-24.408	28.280	0.410	-79.838	31.021	-25.796	40.454	0.540	-105.086	53.494
HOMA-B	paracentral	frontal	10	-13.533	25.704	0.599	-63.912	36.846	-27.728	85.618	0.754	-195.539	140.083	-10.355	27.910	0.711	-65.059	44.349	-10.792	29.160	0.720	-67.946	46.363	-15.867	39.395	0.697	-93.080	61.346
HOMA-B	parsopercularis	frontal	10	28.497	27.186	0.295	-24.788	81.782	87.973	87.994	0.347	-84.494	260.441	60.933	37.897	0.108	-13.346	135.212	88.736	52.679	0.126	-14.515	191.988	77.207	59.609	0.227	-39.627	194.040
HOMA-B	parsorbitalis	frontal	10	5.083	9.063	0.575	-12.680	22.847	19.005	28.521	0.524	-36.896	74.905	7.603	12.045	0.528	-16.005	31.211	6.440	13.357	0.641	-19.740	32.620	2.140	18.070	0.908	-33.277	37.558
HOMA-B	parstriangularis	frontal	10	42.402	29.981	0.157	-16.362	101.165	80.417	99.071	0.440	-113.763	274.597	69.178	32.009	0.031	6.440	131.916	79.735	39.324	0.073	2.660	156.810	84.528	50.229	0.127	-13.921	182.977
HOMA-B	precentral	frontal	10	-21.825	47.897	0.649	-115.703	72.052	124.526	150.764	0.433	-170.972	420.024	-14.068	65.114	0.829	-141.691	113.555	-0.346	81.547	0.997	-160.178	159.485	61.775	101.333	0.557	-136.837	260.387
HOMA-B	rostral anterior cingulate	frontal	10	-2.409	14.539	0.868	-30.905	26.087	-14.751	48.311	0.768	-109.441	79.939	3.504	20.191	0.862	-36.071	43.079	-21.732	27.467	0.449	-75.567	32.102	20.975	34.698	0.560	-47.033	88.984
HOMA-B	rostral middle frontal	frontal	10	-47.747	60.063	0.427	-165.470	69.977	-103.146	189.420	0.601	-474.409	268.117	-110.688	79.255	0.163	-266.027	44.651	-134.349	103.386	0.226	-336.985	68.288	-152.471	141.809	0.310	-430.416	125.474
HOMA-B	superior frontal	frontal	10	9.446	61.657	0.878	-111.402	130.293	12.121	205.833	0.954	-391.312	415.554	-5.368	80.340	0.947	-162.834	152.099	-36.099	99.541	0.725	-231.199	159.001	-123.872	135.407	0.384	-389.269	141.525
HOMA-B	bankssts	temporal	10	14.967	17.184	0.384	-18.714	48.648	-25.320	55.377	0.660	-133.859	83.220	10.969	22.451	0.625	-33.035	54.973	-15.329	30.273	0.625	-74.664	44.006	31.046	35.516	0.405	-38.567	100.658
HOMA-B	entorhinal	temporal	10	-3.658	9.555	0.702	-22.385	15.069	-10.606	30.075	0.733	-69.553	48.341	-5.249	11.610	0.651	-28.005	17.507	-4.339	14.539	0.772	-32.836	24.158	-3.706	17.369	0.836	-37.749	30.338
HOMA-B	fusiform	temporal	10	47.864	45.355	0.291	-41.031	136.759	148.183	146.707	0.342	-139.362	435.728	63.985	49.106	0.193	-32.262	160.233	64.970	51.920	0.242	-36.794	166.733	57.086	72.270	0.450	-84.563	198.736
HOMA-B	inferior temporal	temporal	10	-70.163	41.501	0.091	-151.506	11.179	-93.046	130.594	0.496	-349.011	162.919	-105.533	53.953	0.050	-211.281	0.214	-131.291	74.277	0.111	-276.875	14.293	-120.042	89.261	0.212	-294.994	54.909
HOMA-B	insula	temporal	10	55.141	39.572	0.163	-22.421	132.703	48.284	132.099	0.724	-210.630	307.199	2.933	36.093	0.935	-67.808	73.675	-10.931	43.574	0.808	-96.337	74.474	-17.816	57.427	0.763	-130.372	94.741
HOMA-B	middle temporal	temporal	10	-20.141	43.223	0.641	-104.859	64.576	215.924	114.332	0.096	-8.167	440.014	48.256	49.661	0.331	-49.080	145.592	60.483	56.390	0.311	-50.041	171.007	64.069	90.883	0.499	-114.061	242.200
HOMA-B	parahippocampal	temporal	10	-16.523	13.877	0.234	-43.722	10.676	12.354	45.056	0.791	-75.957	100.664	-7.882	15.059	0.601	-37.398	21.634	-1.734	16.181	0.917	-33.450	29.981	3.453	23.378	0.886	-42.369	49.274
HOMA-B	superior temporal	temporal	10	35.188	34.582	0.309	-32.591	102.968	37.232	110.318	0.744	-178.991	253.455	33.956	45.548	0.456	-55.318	123.230	41.559	49.938	0.427	-56.320	139.438	27.643	68.779	0.697	-107.164	162.450
HOMA-B	temporal pole	temporal	10	-11.215	7.108	0.115	-25.146	2.716	-4.101	22.365	0.859	-47.936	39.734	-10.619	8.810	0.228	-27.887	6.649	-8.515	11.033	0.460	-30.140	13.110	-13.747	14.564	0.370	-42.293	14.798
HOMA-B	transverse temporal	temporal	10	7.383	7.292	0.311	-6.908	21.675	16.576	22.947	0.491	-28.400	61.552	7.406	9.279	0.425	-10.781	25.593	6.526	11.720	0.591	-16.445	29.496	1.036	15.052	0.947	-28.467	30.539
HOMA-B	inferior parietal	parietal	10	-51.768	70.294	0.461	-189.545	86.009	178.088	218.368	0.438	-249.914	606.090	-9.744	81.804	0.905	-170.080	150.591	-5.770	94.801	0.953	-191.580	180.040	-103.677	134.330	0.460	-366.963	159.609
HOMA-B	isthmus cingulate	parietal	10	13.911	20.642	0.500	-26.546	54.369	-40.154	65.870	0.559	-169.259	88.952	9.375	23.618	0.691	-36.916	55.666	3.691	27.097	0.895	-49.421	56.802	5.792	35.405	0.874	-63.601	75.185
HOMA-B	postcentral	parietal	10	-27.524	44.479	0.536	-114.702	59.654	-61.276	147.996	0.690	-351.348	228.795	-57.339	55.604	0.302	-166.323	51.644	-65.851	73.470	0.393	-209.851	78.149	-82.415	99.218	0.428	-276.882	112.051
HOMA-B	posterior cingulate	parietal	10	12.861	18.042	0.476	-22.501	48.224	71.608	56.106	0.238	-38.360	181.576	29.126	21.519	0.176	-13.052	71.304	32.332	22.588	0.186	-11.940	76.603	30.819	30.078	0.332	-28.135	89.772
HOMA-B	precuneus	parietal	10	2.013	40.883	0.961	-78.118	82.144	92.257	128.713	0.494	-160.019	344.534	27.069	55.029	0.623	-80.789	134.927	39.329	60.770	0.534	-79.780	158.439	63.478	78.727	0.441	-90.826	217.782
HOMA-B	superior parietal	parietal	10	-51.906	59.041	0.379	-167.626	63.814	-221.577	186.220	0.268	-586.567	143.414	-78.758	78.857	0.318	-233.317	75.801	-111.104	94.197	0.268	-295.729	73.521	-53.991	127.265	0.681	-303.430	195.449
HOMA-B	supramarginal	parietal	10	80.154	47.881	0.094	-13.694	174.001	153.762	150.690	0.337	-141.590	449.114	88.455	59.409	0.137	-27.987	204.897	111.878	78.987	0.190	-42.936	266.693	152.973	96.361	0.147	-35.895	341.841
HOMA-B	cuneus	occipital	10	9.497	22.573	0.674	-34.745	53.740	5.221	75.334	0.946	-142.434	152.875	-13.923	26.972	0.606	-66.788	38.943	-6.112	31.997	0.853	-68.826	56.602	-24.700	37.299	0.524	-97.805	48.405
HOMA-B	lateral occipital	occipital	10	-31.889	54.759	0.560	-139.217	75.438	-6.447	172.287	0.971	-344.131	331.236	-26.116	73.130	0.721	-169.451	117.219	-11.770	85.674	0.894	-179.691	156.151	46.283	104.763	0.669	-159.052	251.618
HOMA-B	lingual	occipital	10	-36.642	43.158	0.396	-121.231	47.947	-29.551	135.798	0.833	-295.716	236.613	-46.341	60.598	0.444	-165.113	72.430	-132.758	88.183	0.166	-305.597	40.081	-135.977	108.036	0.240	-347.728	75.773
HOMA-B	pericalcarine	occipital	10	-28.859	33.542	0.390	-94.601	36.883	33.116	109.535	0.770	-181.573	247.804	6.361	41.234	0.877	-74.457	87.179	35.012	63.125	0.593	-88.713	158.738	18.128	81.812	0.830	-142.224	178.480
HOMA-IR	whole cortex	global	8	-1513.604	2450.034	0.537	-6315.670	3288.461	-15922.802	17742.538	0.404	-50698.177	18852.573	-269.021	3130.258	0.932	-6404.327	5866.286	739.037	4076.409	0.861	-7250.725	8728.798	1277.368	4451.748	0.782	-7448.059	10002.794
HOMA-IR	caudal anterior cingulate	frontal	8	0.119	21.928	0.996	-42.860	43.098	-345.106	121.422	0.029	-583.093	-107.119	-13.212	23.957	0.581	-60.167	33.743	-23.349	40.613	0.583	-102.951	56.253	-18.709	42.494	0.673	-101.997	64.579
HOMA-IR	caudal middle frontal	frontal	8	-62.975	50.929	0.216	-162.795	36.846	-499.513	354.978	0.209	-1195.269	196.243	-49.600	56.160	0.377	-159.674	60.475	-43.080	69.235	0.554	-178.779	92.620	-39.314	76.302	0.622	-188.866	110.238
HOMA-IR	frontal pole	frontal	8	-3.425	5.421	0.527	-14.050	7.200	-40.524	39.477	0.344	-117.898	36.851	-2.478	7.130	0.728	-16.452	11.496	-1.010	10.342	0.925	-21.281	19.261	-1.010	10.294	0.925	-21.186	19.167
HOMA-IR	lateral orbitofrontal	frontal	8	51.961	38.567	0.178	-23.629	127.552	125.300	300.186	0.691	-463.064	713.664	9.470	44.596	0.832	-77.937	96.877	-10.694	58.982	0.861	-126.299	104.910	-3.883	72.472	0.959	-145.928	138.162
HOMA-IR	medial orbitofrontal	frontal	8	45.776	23.987	0.056	-1.238	92.790	-13.910	173.474	0.939	-353.919	326.098	58.873	31.062	0.058	-2.009	119.755	62.918	42.441	0.182	-20.268	146.103	63.982	48.841	0.232	-31.745	159.710
HOMA-IR	paracentral	frontal	8	23.963	37.519	0.523	-49.575	97.501	-374.503	242.795	0.174	-850.380	101.375	23.554	37.208	0.527	-49.373	96.482	26.210	54.341	0.644	-80.298	132.718	17.977	60.750	0.776	-101.092	137.046
HOMA-IR	parsopercularis	frontal	8	-27.116	35.326	0.443	-96.354	42.123	-124.261	273.123	0.665	-659.582	411.059	-46.250	44.434	0.298	-133.341	40.841	-66.018	65.948	0.350	-195.276	63.240	-86.104	70.833	0.264	-224.937	52.730
HOMA-IR	parsorbitalis	frontal	8	-12.958	10.683	0.225	-33.897	7.980	7.079	77.292	0.930	-144.413	158.572	-27.065	14.455	0.061	-55.397	1.268	-32.145	20.119	0.154	-71.577	7.288	15.423	23.168	0.527	-29.986	60.832
HOMA-IR	parstriangularis	frontal	8	-0.724	33.656	0.983	-66.689	65.242	76.933	261.101	0.778	-434.825	588.692	-8.307	40.786	0.839	-88.249	71.634	63.381	64.211	0.356	-62.472	189.234	-32.284	68.579	0.652	-166.698	102.130
HOMA-IR	precentral	frontal	8	-161.911	56.425	0.004	-272.505	-51.318	122.445	408.704	0.775	-678.614	923.504	-158.881	70.696	0.025	-297.445	-20.316	-148.294	100.458	0.183	-345.190	48.603	-121.108	98.616	0.259	-314.394	72.179
HOMA-IR	rostral anterior cingulate	frontal	8	22.492	16.989	0.186	-10.807	55.791	-111.651	120.554	0.390	-347.936	124.635	39.367	21.677	0.069	-3.120	81.854	40.591	31.288	0.236	-20.734	101.917	41.928	34.227	0.260	-25.158	109.013
HOMA-IR	rostral middle frontal	frontal	8	61.224	70.719	0.387	-77.384	199.833	174.940	511.821	0.744	-828.229	1178.109	97.948	93.444	0.295	-85.203	281.099	103.407	133.750	0.465	-158.742	365.556	93.238	144.823	0.540	-190.615	377.090
HOMA-IR	superior frontal	frontal	8	70.675	77.190	0.360	-80.617	221.966	1200.147	496.409	0.052	227.186	2173.108	41.791	96.398	0.665	-147.150	230.732	-0.786	162.898	0.996	-320.066	318.493	-3.073	160.410	0.985	-317.478	311.331
HOMA-IR	bankssts	temporal	8	6.412	28.051	0.819	-48.567	61.392	-67.410	217.196	0.767	-493.115	358.295	36.693	28.945	0.205	-20.040	93.427	33.638	43.272	0.462	-51.175	118.450	-64.248	58.205	0.306	-178.329	49.833
HOMA-IR	entorhinal	temporal	8	-0.752	11.262	0.947	-22.825	21.321	-130.859	81.526	0.160	-290.650	28.933	-6.297	15.222	0.679	-36.132	23.538	-13.988	24.896	0.592	-62.783	34.808	-11.895	25.596	0.656	-62.064	38.274
HOMA-IR	fusiform	temporal	8	-38.223	53.018	0.471	-142.137	65.692	-466.902	374.717	0.259	-1201.347	267.543	-86.349	58.861	0.142	-201.717	29.019	-135.000	88.079	0.169	-307.634	37.634	-125.659	94.158	0.224	-310.208	58.891
HOMA-IR	inferior temporal	temporal	8	-63.624	58.455	0.276	-178.196	50.948	-663.888	384.012	0.135	-1416.551	88.775	-27.402	68.945	0.691	-162.535	107.731	-26.103	108.609	0.817	-238.977	186.771	24.860	120.099	0.842	-210.534	260.255
HOMA-IR	insula	temporal	8	84.151	29.517	0.004	26.299	142.004	-59.318	213.480	0.790	-477.738	359.103	76.426	39.089	0.051	-0.189	153.041	64.355	62.224	0.335	-57.603	186.313	50.291	64.299	0.460	-75.734	176.316
HOMA-IR	middle temporal	temporal	8	29.860	41.897	0.476	-52.258	111.978	272.811	303.154	0.403	-321.370	866.992	4.721	55.025	0.932	-103.128	112.570	-15.169	73.612	0.843	-159.449	129.111	-7.148	78.084	0.930	-160.193	145.896
HOMA-IR	parahippocampal	temporal	8	-20.358	15.948	0.202	-51.617	10.901	113.465	111.561	0.348	-105.195	332.124	-14.454	19.063	0.448	-51.818	22.909	17.164	34.719	0.636	-50.885	85.213	-58.085	37.630	0.167	-131.840	15.670
HOMA-IR	superior temporal	temporal	8	37.005	53.226	0.487	-67.319	141.329	550.967	358.031	0.175	-150.774	1252.709	76.078	55.027	0.167	-31.775	183.932	107.961	67.290	0.153	-23.927	239.850	98.590	82.242	0.270	-62.605	259.784
HOMA-IR	temporal pole	temporal	8	1.397	10.910	0.898	-19.986	22.781	-146.929	60.632	0.052	-265.768	-28.090	6.200	11.725	0.597	-16.780	29.180	7.440	15.607	0.648	-23.150	38.030	8.621	16.998	0.628	-24.694	41.937
HOMA-IR	transverse temporal	temporal	8	-0.048	9.256	0.996	-18.190	18.093	-12.338	72.169	0.870	-153.789	129.112	3.195	11.550	0.782	-19.444	25.834	7.362	15.295	0.645	-22.616	37.340	6.131	16.276	0.718	-25.770	38.033
HOMA-IR	inferior parietal	parietal	8	28.360	108.251	0.793	-183.812	240.532	-75.778	845.278	0.931	-1732.523	1580.968	54.237	109.402	0.620	-160.190	268.664	145.670	180.639	0.447	-208.382	499.723	94.731	204.150	0.657	-305.403	494.865

HOMA-IR	isthmus cingulate	parietal	8	7.414	19.413	0.703	-30.635	45.463	-183.512	140.452	0.239	-458.797	91.774	-6.193	26.550	0.816	-58.232	45.846	-14.594	41.690	0.737	-96.306	67.119	48.821	43.490	0.299	-36.419	134.061
HOMA-IR	postcentral	parietal	8	-58.093	47.460	0.221	-151.115	34.930	-153.547	343.765	0.671	-827.327	520.233	-54.914	61.923	0.375	-176.282	66.455	-54.308	94.885	0.585	-240.283	131.666	-54.308	98.066	0.597	-246.518	137.901
HOMA-IR	posterior cingulate	parietal	8	3.790	19.144	0.843	-33.732	41.312	-152.244	138.527	0.314	-423.756	119.268	10.832	24.586	0.660	-37.357	59.021	1.053	34.547	0.977	-66.659	68.765	-3.734	37.277	0.923	-76.798	69.329
HOMA-IR	precuneus	parietal	8	41.520	49.724	0.404	-55.938	138.979	-549.364	348.703	0.166	-1232.821	134.094	25.039	64.923	0.700	-102.211	152.289	-28.784	101.241	0.784	-227.216	169.649	157.914	107.363	0.185	-52.517	368.346
HOMA-IR	superior parietal	parietal	8	-75.338	69.574	0.279	-211.704	61.027	351.498	503.877	0.512	-636.101	1339.098	-72.233	87.539	0.409	-243.809	99.344	-62.227	114.916	0.605	-287.463	163.009	-76.803	126.106	0.562	-323.971	170.366
HOMA-IR	supramarginal	parietal	8	24.793	56.392	0.660	-85.735	135.321	-739.778	408.133	0.120	-1539.719	60.164	16.238	70.231	0.817	-121.415	153.891	16.668	116.219	0.890	-211.121	244.456	36.836	124.735	0.776	-207.644	281.317
HOMA-IR	cuneus	occipital	8	-48.702	33.445	0.145	-114.254	16.849	318.998	213.162	0.185	-98.800	736.796	-31.473	34.937	0.368	-99.950	37.003	-2.971	57.439	0.960	-115.552	109.610	-13.996	56.737	0.812	-125.201	97.209
HOMA-IR	lateral occipital	occipital	8	-6.341	64.510	0.922	-132.782	120.099	-421.301	466.904	0.402	-1336.432	493.830	-20.870	83.532	0.803	-184.592	142.853	-70.831	126.365	0.593	-318.507	176.846	134.109	135.320	0.355	-131.118	399.336
HOMA-IR	lingual	occipital	8	-70.173	58.845	0.233	-185.508	45.163	580.094	373.776	0.172	-152.506	1312.694	-56.445	66.488	0.396	-186.762	73.871	-48.849	95.335	0.624	-235.705	138.007	-50.727	108.195	0.653	-262.790	161.336
HOMA-IR	pericalcarine	occipital	8	-49.697	46.737	0.288	-141.301	41.907	416.391	310.690	0.229	-192.561	1025.343	-18.817	49.658	0.705	-116.147	78.512	22.730	75.500	0.772	-125.250	170.710	4.044	80.767	0.961	-154.259	162.348
Proinsulin	whole cortex	global	8	-762.450	653.349	0.243	-2043.014	518.114	-1586.921	1578.372	0.354	-4680.531	1506.689	-543.518	909.343	0.550	-2325.830	1238.794	-1719.221	1244.610	0.210	-4158.656	720.214	718.693	1486.782	0.644	-2195.400	3632.786
Proinsulin	caudal anterior cingulate	frontal	8	-2.076	4.481	0.643	-10.859	6.707	-4.015	10.572	0.717	-24.735	16.706	-1.563	6.189	0.801	-13.692	10.567	5.732	9.315	0.558	-12.526	23.990	5.076	9.733	0.618	-14.001	24.152
Proinsulin	caudal middle frontal	frontal	8	13.088	12.192	0.283	-10.808	36.984	18.549	30.957	0.571	-42.127	79.224	13.958	15.391	0.364	-16.209	44.124	12.581	17.620	0.498	-21.953	47.116	5.483	22.758	0.817	-39.124	50.089
Proinsulin	frontal pole	frontal	8	-0.948	1.493	0.525	-3.875	1.979	-0.029	3.783	0.994	-7.442	7.385	-1.425	1.762	0.419	-4.878	2.028	-0.684	2.209	0.766	-5.013	3.645	-0.536	2.561	0.840	-5.555	4.484
Proinsulin	lateral orbitofrontal	frontal	8	-0.550	10.108	0.957	-20.361	19.261	20.827	23.865	0.416	-25.949	67.603	9.592	10.925	0.380	-11.820	31.004	10.675	12.252	0.412	-13.339	34.689	9.979	15.042	0.528	-19.503	39.461
Proinsulin	medial orbitofrontal	frontal	8	-7.565	6.413	0.238	-20.135	5.005	-13.445	16.109	0.436	-45.019	18.128	-4.074	8.841	0.645	-21.403	13.255	-0.068	11.154	0.995	-21.930	21.794	-4.918	13.845	0.733	-32.055	22.219
Proinsulin	paracentral	frontal	8	2.624	11.789	0.824	-20.482	25.730	32.550	26.815	0.270	-20.008	85.108	13.334	9.909	0.178	-6.087	32.755	15.162	14.669	0.336	-13.588	43.913	14.258	21.201	0.523	-27.296	55.812
Proinsulin	parsopercularis	frontal	8	-0.185	8.194	0.982	-16.244	15.874	1.848	19.326	0.927	-36.031	39.727	3.308	10.203	0.746	-16.690	23.305	6.188	14.049	0.673	-21.348	33.724	6.334	14.411	0.674	-21.911	34.579
Proinsulin	parsorbitalis	frontal	8	-0.302	3.911	0.938	-7.968	7.363	1.695	9.922	0.870	-17.752	21.142	-0.254	3.885	0.948	-7.869	7.360	-1.449	4.649	0.764	-10.562	7.663	-1.268	6.168	0.843	-13.358	10.822
Proinsulin	parstriangularis	frontal	8	-4.587	9.263	0.620	-22.742	13.569	-20.328	22.518	0.401	-64.464	23.808	-0.790	11.083	0.943	-22.512	20.933	20.761	20.494	0.345	-19.407	60.928	19.414	23.797	0.441	-27.228	66.057
Proinsulin	precentral	frontal	8	22.532	16.761	0.179	-10.320	55.384	61.373	38.922	0.166	-14.914	137.661	12.769	20.904	0.541	-28.204	53.742	4.555	30.617	0.886	-55.454	64.563	-15.195	34.363	0.672	-82.545	52.156
Proinsulin	rostral anterior cingulate	frontal	8	0.374	5.415	0.945	-10.240	10.989	12.818	12.600	0.348	-11.879	37.515	0.223	5.737	0.969	-11.021	11.467	0.583	7.888	0.943	-14.878	16.044	0.141	8.971	0.988	-17.442	17.723
Proinsulin	rostral middle frontal	frontal	8	-49.284	18.886	0.009	-86.301	-12.268	-107.222	44.528	0.053	-194.497	-19.947	-57.667	23.478	0.014	-103.684	-11.649	-61.290	29.616	0.077	-119.338	-3.242	-57.337	34.292	0.138	-124.549	9.876
Proinsulin	superior frontal	frontal	8	12.543	19.576	0.522	-25.826	50.912	15.477	49.822	0.767	-82.173	113.128	11.212	23.068	0.627	-34.001	56.425	10.932	26.962	0.697	-41.914	63.778	12.409	34.619	0.731	-55.444	80.262
Proinsulin	bankssts	temporal	8	-0.194	7.016	0.978	-13.945	13.557	-16.763	16.259	0.342	-48.630	15.105	-6.781	6.695	0.311	-19.903	6.341	-7.875	7.532	0.331	-22.638	6.889	-9.801	9.091	0.317	-27.618	8.017
Proinsulin	entorhinal	temporal	8	-3.316	3.005	0.270	-9.205	2.573	-1.536	7.093	0.836	-15.438	12.365	-1.207	3.887	0.756	-8.826	6.411	-2.109	4.387	0.645	-10.708	6.490	-1.034	5.685	0.861	-12.177	10.108
Proinsulin	fusiform	temporal	8	17.109	11.427	0.134	-5.288	39.507	-6.172	26.937	0.826	-58.968	46.624	6.349	15.044	0.673	-23.137	35.834	4.993	17.267	0.781	-28.850	38.836	-3.877	21.390	0.861	-45.802	38.048
Proinsulin	inferior temporal	temporal	8	7.377	13.040	0.572	-18.181	32.936	-33.785	30.735	0.314	-94.026	26.456	-0.705	16.353	0.966	-32.757	31.348	-5.002	21.163	0.820	-46.480	36.477	-1.121	25.922	0.967	-51.928	49.686
Proinsulin	insula	temporal	8	0.432	7.863	0.956	-14.980	15.844	7.729	18.542	0.691	-28.613	44.071	3.036	9.518	0.750	-15.619	21.691	2.379	11.077	0.836	-19.332	24.090	3.005	13.558	0.831	-23.568	29.578
Proinsulin	middle temporal	temporal	8	11.167	11.181	0.318	-10.748	33.082	-20.877	26.364	0.459	-72.550	30.796	4.926	14.753	0.738	-23.990	33.842	-2.113	17.241	0.906	-35.907	31.680	2.951	23.383	0.903	-42.878	48.781
Proinsulin	parahippocampal	temporal	8	2.110	5.442	0.698	-8.557	12.777	5.466	13.800	0.706	-21.581	32.514	1.457	4.701	0.757	-7.756	10.671	-0.040	5.137	0.994	-10.109	10.030	-0.955	6.401	0.886	-13.502	11.591
Proinsulin	superior temporal	temporal	8	-2.753	10.859	0.800	-24.036	18.530	-6.004	25.608	0.822	-56.195	44.187	-8.472	13.412	0.528	-34.759	17.815	-11.109	17.022	0.535	-44.473	22.254	-9.278	20.214	0.660	-48.898	30.342
Proinsulin	temporal pole	temporal	8	3.400	2.235	0.128	-0.980	7.780	3.643	5.273	0.515	-6.692	13.979	4.734	2.802	0.091	-0.758	10.226	5.206	3.621	0.194	-1.890	12.302	4.920	4.219	0.282	-3.350	13.189
Proinsulin	transverse temporal	temporal	8	0.931	2.873	0.746	-4.700	6.562	-0.139	7.306	0.985	-14.460	14.181	3.193	3.420	0.350	-3.509	9.895	7.143	5.461	0.232	-3.560	17.847	7.523	6.052	0.254	-4.338	19.385
Proinsulin	inferior parietal	parietal	8	38.031	19.457	0.051	-0.103	76.166	11.302	47.983	0.822	-82.745	105.349	29.550	26.074	0.257	-21.555	80.655	20.122	35.479	0.588	-49.416	89.660	28.139	42.482	0.529	-55.126	111.404
Proinsulin	isthmus cingulate	parietal	8	3.781	5.937	0.524	-7.855	15.417	8.738	14.954	0.580	-20.572	38.048	2.944	6.601	0.656	-9.994	15.881	2.871	7.153	0.700	-11.148	16.890	3.455	8.611	0.700	-13.423	20.333
Proinsulin	postcentral	parietal	8	-8.543	15.601	0.584	-39.120	22.035	-7.151	39.725	0.863	-85.011	70.710	-17.006	17.428	0.329	-51.164	17.153	-16.316	21.956	0.482	-59.349	26.717	-26.073	26.636	0.360	-78.278	26.133
Proinsulin	posterior cingulate	parietal	8	5.254	6.867	0.444	-8.204	18.713	-10.353	16.014	0.542	-41.740	21.034	8.155	7.609	0.284	-6.758	23.069	18.714	15.881	0.277	-12.413	49.840	15.625	15.047	0.334	-13.866	45.117
Proinsulin	precuneus	parietal	8	11.091	12.849	0.388	-14.094	36.276	2.570	30.304	0.935	-56.826	61.966	10.777	15.987	0.500	-20.557	42.110	-3.144	22.092	0.891	-46.443	40.156	17.695	25.548	0.511	-32.379	67.769
Proinsulin	superior parietal	parietal	8	-2.172	18.549	0.907	-38.528	34.185	-28.090	43.748	0.545	-113.835	57.655	-3.282	26.280	0.901	-54.791	48.227	2.760	36.460	0.942	-68.702	74.223	0.722	41.037	0.986	-79.710	81.153
Proinsulin	supramarginal	parietal	8	-2.829	17.885	0.874	-37.884	32.227	17.152	44.664	0.714	-70.389	104.692	-11.681	18.689	0.532	-48.312	24.949	-4.335	21.418	0.845	-46.315	37.645	-7.058	26.674	0.799	-59.338	45.223
Proinsulin	cuneus	occipital	8	-14.917	6.687	0.026	-28.025	-1.810	-6.798	16.627	0.697	-39.386	25.790	-19.212	8.905	0.031	-36.665	-1.759	-22.817	15.077	0.174	-52.368	6.735	-18.942	15.987	0.275	-50.278	12.393
Proinsulin	lateral occipital	occipital	8	0.857	17.200	0.960	-32.855	34.570	-11.718	40.560	0.782	-91.216	67.781	-1.543	20.678	0.940	-42.072	38.985	2.229	25.029	0.932	-46.828	51.286	0.357	28.739	0.990	-55.971	56.685
Proinsulin	lingual	occipital	8	-7.595	13.551	0.575	-34.155	18.964	-3.148	31.972	0.925	-65.813	59.518	-2.796	17.023	0.870	-36.162	30.570	-6.842	21.277	0.757	-48.545	34.861	-29.322	24.710	0.274	-77.753	19.109
Proinsulin	pericalcarine	occipital	8	-11.952	9.020	0.185	-29.631	5.728	-5.645	21.274	0.800	-47.343	36.053	-8.204	11.265	0.466	-30.283	13.876	-4.232	14.433	0.778	-32.521	24.056	-3.428	15.621	0.833	-34.045	27.189

Notes: MR, Mendelian randomization; IVW, inverse variance weighted; IVs, instrumental variables; β, estimate coefficient; SE, standard error of coefficient estimate; CI, confidence interval; HOMA-B, homeostasis model assessment beta-cell function; HOMA-IR, homeostasis model assessment insulin resistance.

Supplementary Table S6. MR analysis results of insulin resistance on cortical thickness.

Causal effects were estimated using five two-sample MR methods (IVW, MR Egger, weighted median, weighted mode, and simple mode). P value < 0.05 was regarded as nominally significant. P value < 0.0015 was considered significant.

Exposure	Outcome	Lobe	IVs N	IVW					MR Egger					Weighted Median					Weighted Mode					Simple Mode				
				Estimate (β)	SE	*P* value	95% lower C	95% upper C	Estimate (β)	SE	*P* value	95% lower C	95% upper C	Estimate (β	SE	*P* value	95% lower C	95% upper C	Estimate (β	SE	*P* value	95% lower C	95% upper C	Estimate (β	SE	*P* value	95% lower C	95% upper CI
Fasting insulin	whole cortex	global	9	-0.003	0.024	0.906	-0.050	0.044	0.074	0.143	0.619	-0.206	0.355	0.032	0.023	0.170	-0.014	0.078	0.037	0.029	0.230	-0.019	0.094	0.035	0.032	0.306	-0.028	0.097
Fasting insulin	caudal anterior cingulate	frontal	9	-0.052	0.035	0.134	-0.120	0.016	-0.080	0.198	0.698	-0.467	0.307	-0.068	0.045	0.128	-0.155	0.020	-0.091	0.067	0.209	-0.222	0.040	-0.076	0.070	0.312	-0.214	0.062
Fasting insulin	caudal middle frontal	frontal	9	0.014	0.017	0.415	-0.019	0.046	-0.003	0.096	0.979	-0.190	0.185	0.011	0.022	0.608	-0.031	0.054	0.002	0.033	0.946	-0.062	0.067	-0.003	0.035	0.933	-0.071	0.065
Fasting insulin	frontal pole	frontal	9	0.039	0.039	0.320	-0.038	0.115	0.046	0.224	0.843	-0.392	0.485	0.073	0.052	0.161	-0.029	0.175	0.085	0.073	0.277	-0.058	0.229	0.083	0.081	0.338	-0.076	0.242
Fasting insulin	lateral orbitofrontal	frontal	9	0.008	0.027	0.771	-0.045	0.060	-0.147	0.153	0.368	-0.446	0.153	0.001	0.030	0.969	-0.057	0.059	-0.027	0.042	0.548	-0.110	0.056	-0.029	0.049	0.575	-0.124	0.067
Fasting insulin	medial orbitofrontal	frontal	9	-0.033	0.031	0.288	-0.095	0.028	0.023	0.191	0.908	-0.351	0.397	-0.021	0.035	0.554	-0.089	0.047	-0.015	0.060	0.804	-0.132	0.102	-0.009	0.059	0.889	-0.125	0.108
Fasting insulin	paracentral	frontal	9	0.009	0.030	0.752	-0.049	0.068	0.175	0.168	0.334	-0.155	0.504	0.030	0.030	0.323	-0.030	0.090	0.080	0.060	0.215	-0.037	0.198	0.082	0.064	0.238	-0.044	0.207
Fasting insulin	parsopercularis	frontal	9	0.009	0.017	0.584	-0.024	0.043	-0.017	0.097	0.863	-0.208	0.173	0.017	0.022	0.434	-0.026	0.060	0.022	0.033	0.516	-0.042	0.087	0.024	0.032	0.468	-0.038	0.086
Fasting insulin	parsorbitalis	frontal	9	0.045	0.027	0.088	-0.007	0.097	0.146	0.152	0.368	-0.152	0.444	0.039	0.035	0.265	-0.030	0.108	0.020	0.054	0.717	-0.086	0.126	0.012	0.053	0.829	-0.093	0.117
Fasting insulin	parstriangularis	frontal	9	0.027	0.018	0.145	-0.009	0.062	0.155	0.104	0.181	-0.049	0.360	0.030	0.025	0.230	-0.019	0.078	0.065	0.039	0.140	-0.013	0.142	-0.009	0.043	0.832	-0.094	0.075
Fasting insulin	precentral	frontal	9	0.036	0.020	0.078	-0.004	0.076	0.034	0.125	0.791	-0.210	0.279	0.046	0.023	0.047	0.001	0.092	0.051	0.042	0.255	-0.031	0.132	0.052	0.040	0.230	-0.027	0.131
Fasting insulin	rostral anterior cingulate	frontal	9	-0.080	0.035	0.022	-0.148	-0.012	-0.180	0.210	0.420	-0.592	0.232	-0.083	0.045	0.063	-0.171	0.004	-0.089	0.060	0.175	-0.206	0.028	-0.086	0.066	0.232	-0.216	0.044
Fasting insulin	rostral middle frontal	frontal	9	0.020	0.015	0.177	-0.009	0.048	0.078	0.083	0.377	-0.084	0.241	0.025	0.018	0.178	-0.011	0.061	0.038	0.027	0.201	-0.015	0.091	0.033	0.028	0.278	-0.023	0.088
Fasting insulin	superior frontal	frontal	9	-0.010	0.015	0.506	-0.039	0.019	-0.144	0.084	0.129	-0.308	0.020	-0.019	0.019	0.339	-0.057	0.020	-0.027	0.028	0.370	-0.082	0.028	-0.021	0.029	0.482	-0.077	0.035
Fasting insulin	bankssts	temporal	9	-0.009	0.022	0.665	-0.051	0.033	0.013	0.123	0.919	-0.227	0.253	-0.009	0.028	0.740	-0.063	0.045	0.018	0.040	0.655	-0.059	0.096	-0.017	0.042	0.702	-0.099	0.066
Fasting insulin	entorhinal	temporal	9	-0.067	0.062	0.283	-0.188	0.055	-0.316	0.367	0.417	-1.035	0.403	-0.137	0.078	0.078	-0.288	0.015	-0.179	0.137	0.227	-0.447	0.089	-0.167	0.142	0.273	-0.446	0.111
Fasting insulin	fusiform	temporal	9	0.000	0.020	0.985	-0.039	0.038	0.097	0.114	0.422	-0.126	0.320	-0.019	0.024	0.417	-0.066	0.027	-0.015	0.036	0.693	-0.085	0.056	-0.012	0.036	0.747	-0.084	0.059
Fasting insulin	inferior temporal	temporal	9	-0.024	0.020	0.235	-0.063	0.015	0.026	0.121	0.837	-0.212	0.263	-0.019	0.028	0.489	-0.073	0.035	-0.013	0.038	0.742	-0.088	0.062	-0.020	0.039	0.612	-0.096	0.055
Fasting insulin	insula	temporal	9	-0.009	0.034	0.796	-0.074	0.057	-0.089	0.202	0.675	-0.485	0.308	0.025	0.034	0.463	-0.042	0.093	0.061	0.057	0.319	-0.051	0.173	0.070	0.061	0.285	-0.050	0.191
Fasting insulin	middle temporal	temporal	9	0.037	0.022	0.096	-0.007	0.080	0.249	0.106	0.052	0.041	0.458	0.029	0.026	0.260	-0.022	0.080	0.058	0.044	0.221	-0.028	0.143	0.067	0.043	0.162	-0.018	0.152
Fasting insulin	parahippocampal	temporal	9	-0.104	0.046	0.024	-0.194	-0.014	-0.056	0.279	0.847	-0.601	0.490	-0.132	0.061	0.031	-0.251	-0.012	-0.144	0.092	0.157	-0.324	0.037	-0.145	0.094	0.162	-0.330	0.040
Fasting insulin	superior temporal	temporal	9	-0.012	0.018	0.503	-0.048	0.024	0.033	0.105	0.764	-0.173	0.238	-0.012	0.025	0.634	-0.060	0.037	-0.006	0.043	0.898	-0.091	0.079	0.006	0.042	0.896	-0.077	0.088
Fasting insulin	temporal pole	temporal	9	-0.040	0.066	0.543	-0.169	0.089	0.210	0.392	0.609	-0.558	0.978	-0.049	0.073	0.500	-0.191	0.093	-0.116	0.116	0.345	-0.343	0.111	0.044	0.132	0.747	-0.215	0.303
Fasting insulin	transverse temporal	temporal	9	-0.018	0.039	0.651	-0.094	0.059	0.184	0.225	0.440	-0.257	0.625	-0.021	0.043	0.636	-0.106	0.065	-0.015	0.068	0.825	-0.148	0.117	-0.017	0.065	0.800	-0.144	0.110
Fasting insulin	inferior parietal	parietal	9	-0.006	0.013	0.673	-0.031	0.020	0.019	0.075	0.805	-0.128	0.166	0.000	0.018	0.988	-0.035	0.035	0.009	0.026	0.750	-0.042	0.059	0.009	0.027	0.758	-0.044	0.061
Fasting insulin	isthmus cingulate	parietal	9	-0.032	0.030	0.286	-0.090	0.026	-0.031	0.170	0.860	-0.365	0.303	-0.038	0.039	0.322	-0.114	0.037	-0.042	0.054	0.465	-0.148	0.065	-0.040	0.057	0.509	-0.152	0.073
Fasting insulin	postcentral	parietal	9	0.002	0.015	0.870	-0.027	0.032	0.015	0.087	0.870	-0.156	0.185	0.004	0.020	0.835	-0.034	0.043	0.001	0.031	0.976	-0.061	0.063	0.001	0.031	0.976	-0.060	0.062
Fasting insulin	posterior cingulate	parietal	9	-0.020	0.028	0.467	-0.075	0.034	0.008	0.170	0.962	-0.325	0.342	-0.034	0.031	0.272	-0.094	0.026	-0.047	0.066	0.496	-0.178	0.083	-0.052	0.056	0.378	-0.162	0.058
Fasting insulin	precuneus	parietal	9	0.018	0.022	0.401	-0.024	0.060	0.008	0.134	0.955	-0.255	0.271	0.039	0.019	0.037	0.002	0.075	0.048	0.024	0.076	0.002	0.094	0.049	0.025	0.086	0.000	0.099
Fasting insulin	superior parietal	parietal	9	0.011	0.020	0.585	-0.028	0.049	0.026	0.118	0.833	-0.206	0.258	0.005	0.020	0.820	-0.035	0.044	0.007	0.026	0.797	-0.044	0.058	0.010	0.034	0.764	-0.055	0.076
Fasting insulin	supramarginal	parietal	9	0.013	0.013	0.316	-0.013	0.039	0.161	0.075	0.070	0.013	0.308	0.020	0.017	0.259	-0.014	0.053	0.032	0.026	0.255	-0.019	0.084	0.036	0.028	0.235	-0.019	0.090
Fasting insulin	cuneus	occipital	9	-0.010	0.019	0.606	-0.047	0.027	-0.093	0.110	0.425	-0.308	0.122	-0.013	0.025	0.583	-0.062	0.035	-0.039	0.042	0.378	-0.121	0.043	-0.039	0.046	0.419	-0.129	0.051
Fasting insulin	lateral occipital	occipital	9	-0.027	0.019	0.165	-0.065	0.011	0.051	0.116	0.672	-0.175	0.277	-0.035	0.021	0.107	-0.077	0.008	-0.033	0.037	0.401	-0.106	0.040	-0.032	0.039	0.430	-0.109	0.044
Fasting insulin	lingual	occipital	9	-0.025	0.021	0.241	-0.066	0.017	0.181	0.102	0.119	-0.019	0.381	-0.022	0.023	0.345	-0.068	0.024	-0.016	0.038	0.686	-0.090	0.058	-0.028	0.038	0.484	-0.104	0.047
Fasting insulin	pericalcarine	occipital	9	-0.041	0.019	0.028	-0.078	-0.004	0.032	0.106	0.770	-0.175	0.240	-0.033	0.023	0.155	-0.078	0.012	-0.030	0.036	0.428	-0.102	0.041	-0.026	0.036	0.496	-0.097	0.045
HOMA-B	whole cortex	global	10	-0.012	0.022	0.592	-0.056	0.032	0.054	0.071	0.462	-0.084	0.193	0.008	0.017	0.665	-0.027	0.042	0.011	0.018	0.550	-0.025	0.047	0.011	0.024	0.647	-0.036	0.058
HOMA-B	caudal anterior cingulate	frontal	10	0.049	0.044	0.269	-0.038	0.136	-0.063	0.142	0.671	-0.341	0.216	0.040	0.043	0.342	-0.043	0.124	0.030	0.052	0.579	-0.071	0.131	0.114	0.088	0.227	-0.058	0.287
HOMA-B	caudal middle frontal	frontal	10	0.019	0.014	0.165	-0.008	0.045	0.008	0.043	0.854	-0.076	0.092	0.030	0.018	0.104	-0.006	0.066	0.040	0.027	0.173	-0.013	0.093	0.041	0.031	0.219	-0.020	0.101
HOMA-B	frontal pole	frontal	10	0.029	0.032	0.368	-0.034	0.091	-0.010	0.101	0.923	-0.207	0.187	0.030	0.043	0.490	-0.055	0.114	0.024	0.052	0.649	-0.077	0.126	0.052	0.068	0.469	-0.082	0.185
HOMA-B	lateral orbitofrontal	frontal	10	-0.003	0.026	0.910	-0.053	0.047	-0.047	0.083	0.588	-0.210	0.116	-0.015	0.023	0.508	-0.060	0.030	-0.021	0.026	0.442	-0.071	0.030	-0.007	0.034	0.842	-0.073	0.059
HOMA-B	medial orbitofrontal	frontal	10	-0.012	0.028	0.663	-0.067	0.042	0.003	0.093	0.971	-0.179	0.186	-0.016	0.026	0.534	-0.066	0.034	-0.018	0.028	0.550	-0.073	0.038	-0.020	0.035	0.583	-0.088	0.049
HOMA-B	paracentral	frontal	10	0.001	0.019	0.970	-0.036	0.037	0.029	0.061	0.646	-0.091	0.150	-0.001	0.020	0.975	-0.039	0.038	0.003	0.021	0.902	-0.039	0.044	-0.010	0.031	0.759	-0.070	0.051
HOMA-B	parsopercularis	frontal	10	0.002	0.014	0.870	-0.025	0.029	0.001	0.043	0.981	-0.083	0.085	0.001	0.018	0.973	-0.035	0.036	-0.006	0.021	0.790	-0.047	0.035	-0.013	0.028	0.652	-0.069	0.042
HOMA-B	parsorbitalis	frontal	10	-0.011	0.025	0.676	-0.060	0.039	-0.122	0.075	0.141	-0.268	0.024	-0.052	0.031	0.089	-0.112	0.008	-0.061	0.039	0.150	-0.137	0.015	-0.062	0.057	0.304	-0.174	0.050
HOMA-B	parstriangularis	frontal	10	-0.030	0.020	0.136	-0.070	0.010	-0.107	0.062	0.125	-0.229	0.015	-0.022	0.022	0.332	-0.066	0.022	-0.026	0.029	0.393	-0.081	0.030	-0.039	0.038	0.336	-0.113	0.036
HOMA-B	precentral	frontal	10	-0.010	0.013	0.453	-0.036	0.016	0.037	0.043	0.415	-0.047	0.120	0.001	0.017	0.961	-0.033	0.035	0.003	0.020	0.901	-0.037	0.042	0.002	0.026	0.951	-0.050	0.053
HOMA-B	rostral anterior cingulate	frontal	10	-0.004	0.032	0.899	-0.066	0.058	0.027	0.106	0.806	-0.181	0.234	0.006	0.036	0.872	-0.064	0.076	0.013	0.040	0.757	-0.065	0.090	0.042	0.062	0.511	-0.079	0.164
HOMA-B	rostral middle frontal	frontal	10	-0.001	0.012	0.958	-0.024	0.023	-0.051	0.038	0.218	-0.125	0.024	-0.006	0.016	0.724	-0.037	0.026	-0.013	0.021	0.549	-0.053	0.028	-0.010	0.028	0.734	-0.064	0.045
HOMA-B	superior frontal	frontal	10	0.004	0.012	0.718	-0.019	0.028	0.026	0.038	0.510	-0.048	0.101	0.011	0.016	0.479	-0.020	0.042	0.013	0.018	0.502	-0.023	0.049	0.010	0.024	0.698	-0.038	0.057
HOMA-B	bankssts	temporal	10	-0.014	0.020	0.486	-0.053	0.025	-0.073	0.063	0.275	-0.197	0.050	-0.023	0.024	0.347	-0.070	0.024	-0.030	0.029	0.334	-0.087	0.027	-0.031	0.036	0.421	-0.102	0.041
HOMA-B	entorhinal	temporal	10	0.022	0.048	0.645	-0.072	0.117	-0.011	0.160	0.945	-0.325	0.303	0.020	0.061	0.742	-0.100	0.141	0.017	0.072	0.823	-0.125	0.158	0.046	0.095	0.642	-0.141	0.232
HOMA-B	fusiform	temporal	10	-0.007	0.025	0.765	-0.056	0.041	0.008	0.081	0.923	-0.151	0.167	-0.009	0.021	0.656	-0.051	0.032	-0.005	0.025	0.831	-0.054	0.043	-0.077	0.050	0.159	-0.174	0.021
HOMA-B	inferior temporal	temporal	10	-0.007	0.019	0.693	-0.044	0.029	-0.040	0.062	0.536	-0.160	0.081	-0.013	0.025	0.614	-0.062	0.036	-0.055	0.040	0.200	-0.134	0.023	0.043	0.048	0.394	-0.052	0.138
HOMA-B	insula	temporal	10	-0.026	0.020	0.201	-0.066	0.014	-0.105	0.062	0.129	-0.226	0.017	-0.009	0.026	0.737	-0.059	0.042	0.015	0.044	0.736	-0.071	0.101	0.032	0.048	0.522	-0.062	0.126
HOMA-B	middle temporal	temporal	10	0.003	0.019	0.864	-0.033	0.040	-0.041	0.060	0.513	-0.158	0.076	-0.006	0.020	0.787	-0.046	0.034	-0.008	0.023	0.748	-0.052	0.037	0.011	0.028	0.706	-0.044	0.066
HOMA-B	parahippocampal	temporal	10	0.033	0.043	0.444	-0.051	0.116	-0.207	0.119	0.119	-0.440	0.026	-0.024	0.054	0.657	-0.129	0.082	-0.050	0.058	0.412	-0.163	0.064	0.145	0.107	0.209	-0.065	0.356
HOMA-B	superior temporal	temporal	10	-0.007	0.019	0.736	-0.045	0.032	-0.108	0.053	0.076	-0.211	-0.004	-0.036	0.021	0.086	-0.077	0.005	-0.041	0.023	0.108	-0.087	0.004	-0.030	0.039	0.459	-0.106	0.046
HOMA-B	temporal pole	temporal	10	-0.006	0.055	0.913	-0.115	0.103	-0.381	0.125	0.016	-0.626	-0.136	-0.120	0.057	0.036	-0.231	-0.008	-0.132	0.069	0.088	-0.268	0.003	0.116	0.134	0.411	-0.148	0.380
HOMA-B	transverse temporal	temporal	10	-0.039	0.025	0.114	-0.088	0.009	0.027	0.078	0.740	-0.126	0.179	-0.036	0.033	0.271	-0.100	0.028	-0.036	0.041	0.406	-0.116	0.045	-0.068	0.054	0.236	-0.174	0.037
HOMA-B	inferior parietal	parietal	10	0.008	0.011	0.472	-0.013	0.029	0.015	0.033	0.656	-0.050	0.081	0.015	0.033	0.656	-0.050	0.081	0.020	0.015	0.234	-0.011	0.050	0.019	0.020	0.363	-0.020	0.058
HOMA-B	isthmus cingulate	parietal	10	-0.013	0.034	0.701	-0.079	0.053	-0.127	0.103	0.253	-0.330	0.075	-0.011	0.037	0.767	-0.084	0.062	-0.032	0.042	0.469	-0.115	0.051	0.000	0.063	0.998	-0.124	0.124
HOMA-B	postcentral	parietal	10	0.004	0.012	0.735	-0.020	0.028	0.013	0.038	0.743	-0.062	0.088	0.009	0.016	0.565	-0.022	0.041	0.012	0.020	0.557	-0.027	0.051	0.007	0.027	0.798	-0.045	0.059
HOMA-B	posterior cingulate	parietal	10	-0.003	0.022	0.905	-0.045	0.040	-0.081	0.067	0.260	-0.212	0.050	-0.025	0.023	0.283	-0.071	0.021	-0.025	0.026	0.374	-0.076	0.027	-0.025	0.036	0.515	-0.096	0.047
HOMA-B	precuneus	parietal	10	0.017	0.013	0.196	-0.009	0.044	0.039	0.044	0.401	-0.047	0.124	0.033	0.014	0.023	0.004	0.061	0.033	0.017	0.081	0.000	0.066	0.042	0.023	0.103	-0.003	0.088
HOMA-B	superior parietal	parietal	10	0.004	0.011	0.718	-0.017	0.025	0.012	0.034	0.736	-0.054	0.078	0.007	0.013	0.594	-0.019	0.033	0.007	0.016	0.679	-0.025	0.039	-0.001	0.021	0.952	-0.043	0.041
HOMA-B	supramarginal	parietal	10	0.004	0.013	0.763	-0.021	0.029	-0.005	0.042	0.907	-0.087	0.077	0.006	0.015	0.691	-0.023	0.034	0.004	0.017	0.835	-0.029	0.036	-0.003	0.026	0.915	-0.054	0.048
HOMA-B	cuneus	occipital	10	-0.002	0.018	0.904	-0.037	0.033	0.044	0.056	0.461	-0.067	0.154	0.014	0.021	0.489	-0.026	0.055	0.021	0.023	0.379	-0.024	0.066	0.023	0.037	0.549	-0.049	0.094
HOMA-B	lateral occipital	occipital	10	-0.015	0.013	0.247	-0.040	0.010	-0.030	0.042	0.491	-0.113	0.052	-0.017	0.016	0.299	-0.049	0.015	-0.013	0.018	0.475	-0.048	0.022	-0.001	0.026	0.980	-0.051	0.050
HOMA-B	lingual	occipital	10	-0.006	0.017	0.735	-0.038	0.027	0.065	0.049	0.220	-0.031	0.160	0.014	0.019	0.448	-0.022	0.051	0.016	0.020	0.434	-0.023	0.055	0.019	0.027	0.503	-0.035	0.073
HOMA-B	pericalcarine	occipital	10	0.001	0.015	0.973	-0.030	0.031	0.047	0.048	0.358	-0.048	0.142	0.005	0.019	0.806	-0.033	0.043	0.007	0.024	0.782	-0.040	0.054	-0.013	0.029	0.659	-0.071	0.044
HOMA-IR	whole cortex	global	8	-0.003	0.025	0.909	-0.053	0.047	0.221	0.176	0.256	-0.124	0.567	0.033	0.022	0.138	-0.011	0.077	0.040	0.027	0.194	-0.014	0.093	0.042	0.033	0.238	-0.022	0.106
HOMA-IR	caudal anterior cingulate	frontal	8	-0.054	0.033	0.109	-0.119	0.012	-0.151	0.243	0.557	-0.628	0.326	-0.076	0.043	0.077	-0.160	0.008	-0.089	0.059	0.171	-0.204	0.025	-0.084	0.066	0.244	-0.213	0.045
HOMA-IR	caudal middle frontal	frontal	8	0.024	0.016	0.145	-0.008	0.055	-0.009	0.118	0.940	-0.240	0.221	0.029	0.020	0.155	-0.011	0.068	0.042	0.030	0.201	-0.016	0.100	0.040	0.035	0.286	-0.028	0.108
HOMA-IR	frontal pole	frontal	8	0.027	0.038	0.482	-0.047	0.100	0.245	0.275	0.407	-0.294	0.784	0.059	0.049	0.232	-0.038	0.155	0.071	0.066	0.323	-0.060	0.201	0.067	0.077	0.412	-0.084	0.218
HOMA-IR	lateral orbitofrontal	frontal	8	-0.003	0.022	0.898	-0.045	0.040	-0.149	0.159	0.384	-0.461	0.162	-0.003	0.027	0.900	-0.056	0.049	-0.021	0.037	0.589	-0.095	0.052	-0.013	0.040	0.746	-0.092	0.065
HOMA-IR	medial orbitofrontal	frontal	8	-0.039	0.028	0.171	-0.094	0.017	0.126	0.211	0.573	-0.288	0.541	-0.028	0.032	0.382	-0.089	0.034	-0.034	0.050	0.517	-0.131	0.063	-0.022	0.051	0.684	-0.121	0.078
HOMA-IR	paracentral	frontal	8	0.017	0.031	0.595	-0.044	0.078	0.320	0.206	0.171	-0.083	0.723	0.056	0.027	0.040	0.003	0.110	0.071	0.033	0.069	0.006	0.136	0.073	0.038	0.094	-0.001	0.147
HOMA-IR	parsopercularis	frontal	8	0.011	0.016	0.513	-0.021	0.043	-0.066	0.118	0.598	-0.298	0.166	0.018	0.019	0.341	-0.019	0.056	0.021	0.028	0.489	-0.035	0.076	0.019	0.030	0.540	-0.039	0.078
HOMA-IR	parsorbitalis	frontal	8	0.045	0.026	0.078	-0.005	0.096	0.000	0.185	0.999	-0.362	0.362	0.032	0.032	0.321	-0.031	0.095	0.016	0.049	0.746	-0.079	0.112	0.018	0.053	0.740	-0.086	0.122
HOMA-IR	parstriangularis	frontal	8	0.024	0.018	0.183	-0.011	0.059	0.164	0.129	0.252	-0.090	0.418	0.015	0.024	0.520	-0.031	0.062	-0.006	0.037	0.885	-0.079	0.068	0.000	0.039	0.997	-0.076	0.076
HOMA-IR	precentral	frontal	8	0.033	0.019	0.089	-0.005	0.071	0.191	0.139	0.219	-0.082	0.464	0.045	0.022	0.044	0.001	0.088	0.049	0.028	0.130	-0.007	0.104	0.045	0.034	0.229	-0.022	0.113
HOMA-IR	rostral anterior cingulate	frontal	8	-0.093	0.032	0.003	-0.154	-0.031	0.007	0.227	0.975	-0.437	0.452	-0.076	0.038	0.047	-0.151	-0.001	-0.076	0.058	0.229	-0.189	0.037	-0.079	0.059	0.224	-0.194	0.037
HOMA-IR	rostral middle frontal	frontal	8	0.020	0.014	0.154	-0.008	0.048	0.011	0.103	0.922	-0.191	0.212	0.025	0.018	0.168	-0.011	0.061	0.042	0.028	0.179	-0.013	0.097	0.041	0.029	0.202	-0.016	0.098
HOMA-IR	superior frontal	frontal	8	-0.010	0.014	0.491	-0.038	0.018	-0.240	0.103	0.059	-0.442	-0.038	-0.018	0.018	0.312	-0.053	0.017	-0.030	0.028	0.318	-0.085	0.025	-0.031	0.029	0.321	-0.088	0.026
HOMA-IR	bankssts	temporal	8	-0.022	0.021	0.286	-0.063	0.019	0.043	0.151	0.786	-0.254	0.340	-0.010	0.027	0.719	-0.062	0.043	0.004	0.040	0.919	-0.074	0.082	0.004	0.042	0.923	-0.078	0.086
HOMA-IR	entorhinal	temporal	8	-0.100	0.056	0.074	-0.210	0.010	-0.036	0.437	0.937	-0.892	0.820	-0.141	0.068	0.039	-0.275	-0.007	-0.160	0.098	0.146	-0.352	0.032	-0.158	0.110	0.194	-0.374	0.058
HOMA-IR	fusiform	temporal	8	-0.001	0.020	0.960	-0.041	0.039	0.042	0.156	0.796	-0.263	0.347	-0.016	0.023	0.484	-0.060	0.028	-0.021	0.033	0.541	-0.085	0.043	-0.022	0.032	0.503	-0.085	0.040
HOMA-IR	inferior temporal	temporal	8	-0.031	0.019	0.097	-0.068	0.006	0.175	0.135	0.243	-0.090	0.441	-0.016	0.026	0.531	-0.066	0.034	-0.009	0.034	0.797	-0.075	0.057	-0.005	0.040	0.907	-0.083	0.074

HOMA-IR	insula	temporal	8	-0.029	0.031	0.345	-0.091	0.032	0.121	0.238	0.630	-0.346	0.587	0.006	0.032	0.850	-0.056	0.068	0.028	0.046	0.558	-0.062	0.119	0.026	0.057	0.658	-0.085	0.138
HOMA-IR	middle temporal	temporal	8	0.021	0.020	0.300	-0.018	0.060	0.378	0.129	0.027	0.124	0.631	0.022	0.023	0.337	-0.023	0.068	0.029	0.039	0.484	-0.047	0.104	0.032	0.041	0.462	-0.049	0.113
HOMA-IR	parahippocampal	temporal	8	-0.066	0.045	0.137	-0.154	0.021	-0.357	0.325	0.313	-0.993	0.279	-0.102	0.055	0.067	-0.210	0.007	-0.119	0.079	0.177	-0.275	0.037	-0.120	0.083	0.191	-0.282	0.042
HOMA-IR	superior temporal	temporal	8	-0.013	0.018	0.470	-0.048	0.022	0.027	0.129	0.842	-0.227	0.281	-0.026	0.024	0.286	-0.072	0.021	-0.039	0.036	0.319	-0.109	0.032	-0.043	0.037	0.285	-0.115	0.030
HOMA-IR	temporal pole	temporal	8	-0.045	0.071	0.523	-0.185	0.094	0.239	0.547	0.677	-0.832	1.311	-0.125	0.073	0.086	-0.268	0.018	-0.154	0.092	0.138	-0.334	0.026	-0.129	0.104	0.256	-0.334	0.076
HOMA-IR	transverse temporal	temporal	8	-0.029	0.038	0.455	-0.103	0.046	0.128	0.292	0.676	-0.444	0.700	-0.015	0.041	0.716	-0.096	0.066	-0.007	0.078	0.933	-0.160	0.146	-0.011	0.070	0.884	-0.147	0.126
HOMA-IR	inferior parietal	parietal	8	-0.006	0.013	0.660	-0.031	0.019	-0.090	0.092	0.365	-0.270	0.090	0.004	0.017	0.799	-0.029	0.037	0.011	0.025	0.684	-0.039	0.060	0.012	0.025	0.632	-0.036	0.061
HOMA-IR	isthmus cingulate	parietal	8	-0.023	0.029	0.426	-0.080	0.034	0.014	0.210	0.947	-0.398	0.427	-0.040	0.036	0.263	-0.111	0.030	-0.053	0.058	0.390	-0.166	0.060	-0.055	0.056	0.359	-0.166	0.055
HOMA-IR	postcentral	parietal	8	0.004	0.015	0.773	-0.025	0.033	-0.113	0.108	0.333	-0.324	0.098	-0.001	0.019	0.949	-0.039	0.036	-0.003	0.028	0.909	-0.059	0.052	-0.003	0.028	0.908	-0.058	0.051
HOMA-IR	posterior cingulate	parietal	8	-0.012	0.029	0.677	-0.068	0.044	0.083	0.219	0.718	-0.347	0.512	-0.011	0.031	0.731	-0.070	0.049	0.006	0.053	0.914	-0.098	0.110	-0.029	0.056	0.624	-0.138	0.081
HOMA-IR	precuneus	parietal	8	0.019	0.021	0.354	-0.021	0.059	-0.015	0.162	0.931	-0.333	0.304	0.043	0.018	0.019	0.007	0.079	0.047	0.021	0.058	0.006	0.088	0.044	0.022	0.082	0.002	0.087
HOMA-IR	superior parietal	parietal	8	0.018	0.015	0.233	-0.012	0.048	-0.036	0.115	0.765	-0.262	0.190	0.009	0.018	0.602	-0.025	0.043	0.010	0.025	0.692	-0.038	0.059	0.016	0.026	0.569	-0.036	0.067
HOMA-IR	supramarginal	parietal	8	0.014	0.013	0.296	-0.012	0.039	0.142	0.093	0.177	-0.040	0.324	0.022	0.017	0.193	-0.011	0.055	0.038	0.024	0.157	-0.009	0.085	0.039	0.026	0.186	-0.013	0.091
HOMA-IR	cuneus	occipital	8	-0.004	0.018	0.815	-0.040	0.032	0.013	0.141	0.930	-0.264	0.290	-0.006	0.025	0.812	-0.054	0.042	-0.013	0.040	0.758	-0.091	0.066	-0.012	0.039	0.778	-0.089	0.066
HOMA-IR	lateral occipital	occipital	8	-0.014	0.020	0.472	-0.053	0.024	-0.061	0.154	0.707	-0.363	0.242	-0.022	0.022	0.324	-0.065	0.022	-0.026	0.033	0.455	-0.090	0.038	-0.025	0.040	0.544	-0.103	0.052
HOMA-IR	lingual	occipital	8	-0.018	0.021	0.399	-0.060	0.024	0.133	0.157	0.429	-0.175	0.442	-0.015	0.023	0.507	-0.060	0.030	-0.013	0.036	0.727	-0.083	0.057	0.012	0.041	0.786	-0.069	0.092
HOMA-IR	pericalcarine	occipital	8	-0.030	0.018	0.106	-0.066	0.006	0.031	0.131	0.823	-0.226	0.288	-0.018	0.024	0.443	-0.065	0.028	-0.017	0.031	0.613	-0.078	0.045	-0.017	0.035	0.654	-0.086	0.053
Proinsulin	whole cortex	global	8	0.004	0.004	0.407	-0.005	0.012	0.015	0.011	0.206	-0.006	0.036	0.006	0.005	0.237	-0.004	0.017	0.008	0.007	0.301	-0.006	0.021	0.008	0.009	0.382	-0.009	0.025
Proinsulin	caudal anterior cingulate	frontal	8	-0.027	0.009	0.003	-0.044	-0.009	-0.021	0.021	0.360	-0.062	0.020	-0.024	0.012	0.044	-0.047	-0.001	-0.011	0.016	0.520	-0.043	0.021	-0.011	0.020	0.610	-0.050	0.028
Proinsulin	caudal middle frontal	frontal	8	-0.007	0.006	0.248	-0.019	0.005	-0.010	0.016	0.552	-0.040	0.021	-0.008	0.007	0.222	-0.021	0.005	-0.009	0.013	0.505	-0.036	0.017	-0.011	0.012	0.381	-0.034	0.012
Proinsulin	frontal pole	frontal	8	-0.008	0.010	0.446	-0.027	0.012	0.021	0.024	0.419	-0.026	0.067	-0.005	0.012	0.708	-0.029	0.020	-0.006	0.016	0.732	-0.037	0.025	-0.004	0.019	0.826	-0.042	0.033
Proinsulin	lateral orbitofrontal	frontal	8	-0.005	0.010	0.634	-0.025	0.015	-0.001	0.026	0.957	-0.052	0.049	-0.002	0.008	0.839	-0.017	0.014	0.010	0.012	0.444	-0.014	0.034	0.010	0.011	0.410	-0.012	0.031
Proinsulin	medial orbitofrontal	frontal	8	-0.001	0.009	0.880	-0.019	0.016	0.018	0.021	0.415	-0.022	0.058	0.009	0.009	0.323	-0.009	0.026	0.012	0.011	0.326	-0.010	0.034	0.011	0.013	0.417	-0.014	0.036
Proinsulin	paracentral	frontal	8	-0.003	0.007	0.708	-0.016	0.011	0.000	0.017	0.987	-0.034	0.033	0.002	0.007	0.818	-0.013	0.016	0.008	0.010	0.481	-0.012	0.027	0.005	0.013	0.731	-0.022	0.031
Proinsulin	parsopercularis	frontal	8	-0.003	0.005	0.585	-0.012	0.007	-0.005	0.012	0.702	-0.028	0.019	0.003	0.006	0.640	-0.009	0.015	0.004	0.008	0.613	-0.011	0.020	0.005	0.009	0.615	-0.013	0.023
Proinsulin	parsorbitalis	frontal	8	0.000	0.007	0.949	-0.014	0.013	0.017	0.017	0.334	-0.015	0.050	0.002	0.009	0.823	-0.015	0.019	0.005	0.011	0.651	-0.016	0.027	0.008	0.014	0.593	-0.019	0.034
Proinsulin	parstriangularis	frontal	8	0.000	0.005	0.929	-0.010	0.010	0.004	0.013	0.741	-0.021	0.029	0.000	0.006	0.972	-0.012	0.012	-0.005	0.008	0.529	-0.021	0.011	0.010	0.009	0.294	-0.007	0.028
Proinsulin	precentral	frontal	8	-0.003	0.005	0.549	-0.013	0.007	0.001	0.013	0.970	-0.025	0.026	0.000	0.005	1.000	-0.011	0.011	0.000	0.007	0.953	-0.013	0.014	0.003	0.007	0.697	-0.011	0.017
Proinsulin	rostral anterior cingulate	frontal	8	0.010	0.012	0.372	-0.012	0.033	0.040	0.026	0.179	-0.012	0.092	0.002	0.012	0.881	-0.022	0.026	-0.003	0.016	0.831	-0.034	0.027	-0.005	0.022	0.833	-0.047	0.038
Proinsulin	rostral middle frontal	frontal	8	0.000	0.004	0.950	-0.008	0.009	0.014	0.009	0.186	-0.004	0.032	-0.003	0.005	0.570	-0.013	0.007	-0.004	0.008	0.599	-0.019	0.011	-0.006	0.008	0.508	-0.021	0.010
Proinsulin	superior frontal	frontal	8	-0.004	0.004	0.325	-0.012	0.004	-0.007	0.010	0.542	-0.027	0.014	-0.002	0.005	0.680	-0.013	0.008	0.005	0.009	0.575	-0.013	0.023	0.006	0.009	0.535	-0.012	0.024
Proinsulin	bankssts	temporal	8	0.017	0.007	0.013	0.004	0.031	0.001	0.016	0.953	-0.030	0.032	0.009	0.007	0.214	-0.005	0.024	0.009	0.008	0.295	-0.006	0.024	0.008	0.010	0.420	-0.011	0.027
Proinsulin	entorhinal	temporal	8	0.022	0.015	0.139	-0.007	0.050	0.012	0.037	0.758	-0.061	0.084	0.025	0.019	0.188	-0.012	0.062	0.032	0.025	0.241	-0.017	0.082	0.016	0.030	0.613	-0.043	0.074
Proinsulin	fusiform	temporal	8	0.000	0.005	0.922	-0.009	0.009	-0.008	0.011	0.490	-0.030	0.014	-0.006	0.006	0.313	-0.017	0.005	-0.007	0.006	0.293	-0.020	0.005	-0.007	0.008	0.409	-0.023	0.009
Proinsulin	inferior temporal	temporal	8	0.001	0.005	0.822	-0.009	0.011	0.002	0.012	0.881	-0.021	0.025	-0.003	0.006	0.689	-0.015	0.010	-0.003	0.009	0.710	-0.020	0.014	-0.002	0.009	0.829	-0.021	0.016
Proinsulin	insula	temporal	8	0.001	0.006	0.908	-0.011	0.012	0.006	0.014	0.688	-0.022	0.034	-0.002	0.007	0.768	-0.017	0.012	-0.011	0.010	0.316	-0.030	0.009	-0.011	0.011	0.384	-0.033	0.012
Proinsulin	middle temporal	temporal	8	0.008	0.005	0.122	-0.002	0.017	-0.015	0.011	0.232	-0.036	0.007	0.005	0.006	0.466	-0.008	0.017	0.002	0.007	0.778	-0.012	0.016	0.005	0.010	0.610	-0.014	0.024
Proinsulin	parahippocampal	temporal	8	-0.029	0.012	0.014	-0.052	-0.006	-0.036	0.028	0.245	-0.091	0.019	-0.031	0.015	0.038	-0.060	-0.002	-0.026	0.017	0.179	-0.060	0.008	-0.026	0.021	0.262	-0.068	0.016
Proinsulin	superior temporal	temporal	8	0.006	0.005	0.253	-0.004	0.017	-0.008	0.012	0.545	-0.032	0.016	0.005	0.006	0.435	-0.007	0.017	0.003	0.008	0.676	-0.012	0.019	0.004	0.009	0.629	-0.013	0.022
Proinsulin	temporal pole	temporal	8	-0.009	0.015	0.559	-0.037	0.020	-0.024	0.037	0.537	-0.096	0.048	-0.014	0.018	0.438	-0.050	0.022	-0.024	0.027	0.398	-0.076	0.028	-0.017	0.029	0.569	-0.073	0.039
Proinsulin	transverse temporal	temporal	8	0.013	0.008	0.086	-0.002	0.029	0.018	0.018	0.349	-0.017	0.054	0.014	0.010	0.156	-0.005	0.032	0.013	0.012	0.296	-0.010	0.037	0.012	0.014	0.420	-0.015	0.038
Proinsulin	inferior parietal	parietal	8	-0.001	0.003	0.727	-0.008	0.006	-0.004	0.008	0.608	-0.020	0.011	-0.002	0.004	0.614	-0.010	0.006	-0.002	0.005	0.679	-0.011	0.007	-0.003	0.006	0.619	-0.014	0.008
Proinsulin	isthmus cingulate	parietal	8	-0.011	0.015	0.467	-0.040	0.018	0.031	0.033	0.386	-0.034	0.095	0.005	0.012	0.687	-0.018	0.028	0.009	0.014	0.559	-0.019	0.037	-0.002	0.020	0.921	-0.041	0.037
Proinsulin	postcentral	parietal	8	0.000	0.004	0.913	-0.007	0.008	0.001	0.009	0.943	-0.018	0.019	-0.001	0.005	0.876	-0.010	0.009	-0.003	0.007	0.720	-0.017	0.011	-0.003	0.007	0.732	-0.017	0.012
Proinsulin	posterior cingulate	parietal	8	-0.013	0.006	0.015	-0.024	-0.003	-0.005	0.013	0.701	-0.031	0.020	-0.008	0.007	0.246	-0.023	0.006	-0.006	0.009	0.523	-0.024	0.012	-0.006	0.011	0.612	-0.028	0.016
Proinsulin	precuneus	parietal	8	-0.005	0.004	0.154	-0.012	0.002	-0.009	0.009	0.387	-0.027	0.010	-0.006	0.005	0.239	-0.016	0.004	-0.012	0.008	0.185	-0.027	0.004	-0.013	0.009	0.167	-0.030	0.004
Proinsulin	superior parietal	parietal	8	-0.003	0.004	0.447	-0.010	0.004	0.005	0.008	0.605	-0.012	0.021	-0.001	0.004	0.848	-0.010	0.008	0.000	0.005	0.986	-0.010	0.010	-0.001	0.006	0.849	-0.013	0.010
Proinsulin	supramarginal	parietal	8	0.005	0.004	0.168	-0.002	0.012	0.006	0.009	0.500	-0.011	0.023	0.003	0.005	0.459	-0.006	0.013	0.004	0.006	0.488	-0.007	0.015	0.004	0.007	0.602	-0.010	0.017
Proinsulin	cuneus	occipital	8	-0.006	0.005	0.195	-0.016	0.003	-0.001	0.012	0.919	-0.024	0.021	-0.007	0.006	0.217	-0.019	0.004	-0.006	0.007	0.433	-0.020	0.008	-0.006	0.009	0.527	-0.023	0.011
Proinsulin	lateral occipital	occipital	8	0.002	0.004	0.667	-0.006	0.010	0.008	0.010	0.489	-0.012	0.028	-0.002	0.005	0.766	-0.012	0.009	-0.004	0.008	0.645	-0.019	0.012	-0.003	0.009	0.711	-0.021	0.014
Proinsulin	lingual	occipital	8	0.004	0.004	0.345	-0.004	0.012	-0.001	0.010	0.894	-0.021	0.018	0.004	0.005	0.455	-0.007	0.015	0.003	0.006	0.699	-0.010	0.015	0.002	0.007	0.810	-0.013	0.016
Proinsulin	pericalcarine	occipital	8	-0.001	0.005	0.784	-0.011	0.008	-0.014	0.012	0.277	-0.036	0.009	0.002	0.007	0.743	-0.011	0.015	0.003	0.010	0.789	-0.016	0.021	0.004	0.010	0.676	-0.015	0.023

Notes: MR, Mendelian randomization; IVW, inverse variance weighted; IVs, instrumental variables; β, estimate coefficient; SE, standard error of coefficient estimate; CI, confidence interval; HOMA-B, homeostasis model assessment beta-cell function; HOMA-IR, homeostasis model

Supplementary Table S7. Heterogeneity and pleiotropy assessment for the significant forward MR analysis.

Exposure	Outcome	Cochrane's Q test (MR Egger)		Cochrane's Q test (IVW)		MR-PRESSO global test		MR-Egger intercept		
		Q' test	P value	Q' test	P value	RSS_{obs}	P value	Egger_intercept	SE	P value
Fasting insulin	Visual memory	2.260	0.323	2.391	0.495	4.055	0.549	0.003	0.007	0.766
HOMA-B	Visual memory	5.416	0.247	5.601	0.347	7.182	0.420	-0.002	0.004	0.730
Proinsulin	Reaction time	5.599	0.470	5.925	0.548	8.547	0.522	-0.001	0.002	0.588
Fasting insulin	SA of caudal middle frontal	5.673	0.578	5.776	0.672	6.926	0.735	1.752	5.467	0.758
Fasting insulin	SA of lateral orbitofrontal	7.725	0.357	7.727	0.461	9.749	0.499	0.153	4.089	0.971
Fasting insulin	SA of medial orbitofrontal	5.774	0.566	7.351	0.499	9.467	0.499	3.626	2.888	0.250
Fasting insulin	SA of precentral	2.045	0.957	2.717	0.951	3.543	0.950	-5.578	6.805	0.439
Fasting insulin	SA of insula	11.061	0.136	14.383	0.072	17.995	0.090	6.479	4.468	0.190
HOMA-IR	SA of precentral	1.078	0.982	1.572	0.980	2.072	0.983	-6.505	9.259	0.509
HOMA-IR	SA of insula	5.533	0.478	5.993	0.541	7.779	0.570	3.284	4.839	0.523
Proinsulin	SA of rostral middle frontal	2.098	0.910	4.162	0.761	5.326	0.774	5.134	3.573	0.201
Proinsulin	SA of cuneus	6.669	0.353	6.992	0.430	10.252	0.398	-0.720	1.335	0.609
Fasting insulin	TH of rostral anterior cingula	8.927	0.258	9.226	0.324	11.214	0.379	0.002	0.004	0.643
Fasting insulin	TH of parahippocampal	7.870	0.344	7.905	0.443	9.675	0.531	-0.001	0.006	0.865
Fasting insulin	TH of pericalcarine	2.951	0.889	3.445	0.903	4.269	0.928	-0.002	0.002	0.505
HOMA-IR	TH of rostral anterior cingula	3.461	0.749	3.659	0.818	4.549	0.846	-0.002	0.005	0.672
Proinsulin	TH of caudal anterior cingula	5.329	0.502	5.426	0.608	7.848	0.585	-0.001	0.002	0.767
Proinsulin	TH of bankssts	9.014	0.173	10.896	0.143	12.929	0.221	0.001	0.001	0.306
Proinsulin	TH of parahippocampal	4.630	0.592	4.705	0.696	5.606	0.768	0.001	0.002	0.793
Proinsulin	TH of posterior cingulate	5.187	0.520	5.663	0.580	7.449	0.567	-0.001	0.001	0.516
SA of rostralmiddlefrontal	Reaction time	5.879	0.318	6.316	0.389	8.061	0.487	-0.001	0.002	0.569

Notes: MR, Mendelian randomization; IVW, inverse variance weighted; MR-PRESSO, Mendelian randomization pleiotropy residual sum and outlier; RSSobs, observed residual sum of squares; SE, standard error of coefficient estimate; HOMA-B, homeostasis model assessment beta-cell function; HOMA-IR, homeostasis model assessment insulin resistance; SA, surficial area; TH,

Supplementary Table S8. Detailed information of instrumental variables for cognition used in reverse MR analyses (P<5e-

Phenotype	SNP	Effect allele	Other allele	MAF	Beta	SE	P-value	F
Visual memory	rs17385037	C	T	0.306	0.014	0.0025	3.66E-08	29.480
Visual memory	rs11202929	G	A	0.495	0.013	0.0023	4.04E-08	29.206
Visual memory	rs61873589	C	G	0.400	-0.016	0.0024	3.27E-11	42.577
Visual memory	rs11215690	A	A	0.442	-0.014	0.0023	4.54E-09	33.387
Visual memory	rs56002425	T	A	0.220	-0.016	0.0028	6.81E-09	33.641
Visual memory	rs1927551	G	A	0.151	-0.018	0.0032	3.16E-08	29.661
Visual memory	rs1934827	A	T	0.410	-0.015	0.0024	3.10E-10	38.387
Visual memory	rs72774194	A	G	0.135	-0.021	0.0034	7.43E-10	36.730
Visual memory	rs4788190	G	G	0.446	-0.015	0.0023	2.56E-10	38.760
Visual memory	rs11080121	C	T	0.446	0.013	0.0023	2.41E-08	30.193
Visual memory	rs116936671	C	T	0.028	0.044	0.0076	6.31E-09	38.245
Visual memory	rs157592	C	A	0.186	0.017	0.0031	4.89E-08	30.320
Visual memory	rs7582485	C	C	0.395	0.017	0.0024	7.19E-13	50.582
Visual memory	rs12613166	T	A	0.214	0.016	0.0028	3.60E-08	29.488
Visual memory	rs1007876	G	A	0.273	0.015	0.0026	1.38E-08	31.419
Visual memory	rs9866121	A	G	0.195	-0.017	0.0031	3.87E-08	32.453
Visual memory	rs10061099	G	A	0.132	0.019	0.0034	2.51E-08	30.174
Visual memory	rs2195450	A	G	0.248	0.015	0.0027	4.49E-08	29.660
Visual memory	rs191549504	C	T	0.016	0.056	0.0091	5.91E-10	37.164
Visual memory	rs4476937	C	T	0.269	-0.015	0.0026	2.66E-08	30.090
Visual memory	rs199741058	G	G	0.469	-0.013	0.0023	1.36E-08	31.453
Visual memory	rs1149467	G	G	0.239	0.017	0.0027	4.51E-10	38.048
Visual memory	rs10105797	T	T	0.338	-0.015	0.0025	2.03E-09	35.022
Reaction time	rs11205668	C	C	0.435	0.013	0.0022	7.11E-09	29.385
Reaction time	rs61780038	C	C	0.442	0.014	0.0024	8.31E-09	34.384
Reaction time	rs10911301	G	A	0.468	-0.012	0.0022	2.35E-08	27.549
Reaction time	rs1772143	A	T	0.415	0.013	0.0022	3.52E-09	30.628
Reaction time	rs71635159	A	G	0.092	0.022	0.0039	9.73E-09	29.228
Reaction time	rs9429818	T	C	0.262	0.014	0.0025	1.44E-08	28.102
Reaction time	rs72833334	G	A	0.365	0.013	0.0023	9.87E-09	28.966

Reaction time	rs2616612	C	C	0.305	-0.013	0.0024	4.57E-08	26.223
Reaction time	rs7396827	T	T	0.464	-0.013	0.0022	1.64E-08	28.313
Reaction time	rs1484399	G	G	0.420	0.015	0.0022	5.93E-11	37.581
Reaction time	rs4606447	A	A	0.250	-0.019	0.0026	3.76E-13	46.321
Reaction time	rs11603192	G	A	0.369	0.013	0.0023	4.19E-08	26.330
Reaction time	rs10896139	T	C	0.269	0.014	0.0025	1.18E-08	28.763
Reaction time	rs10875906	T	C	0.278	0.018	0.0025	2.61E-12	44.076
Reaction time	rs1029388	C	T	0.211	-0.020	0.0027	1.33E-13	48.031
Reaction time	rs4627212	G	A	0.293	0.017	0.0025	9.31E-12	41.629
Reaction time	rs17568378	C	T	0.185	-0.016	0.0029	1.86E-08	28.313
Reaction time	rs2877960	A	G	0.357	-0.013	0.0023	3.78E-08	26.600
Reaction time	rs16959783	T	C	0.234	0.016	0.0026	7.36E-10	33.403
Reaction time	rs10521240	A	C	0.265	0.014	0.0025	4.26E-08	26.420
Reaction time	rs74776107	G	T	0.083	-0.025	0.0040	5.60E-10	33.990
Reaction time	rs273534	T	T	0.241	0.028	0.0028	6.25E-24	103.047
Reaction time	rs34706544	G	A	0.348	0.014	0.0023	4.44E-09	30.437
Reaction time	rs323299	T	G	0.348	-0.016	0.0023	2.12E-12	43.177
Reaction time	rs4257287	C	C	0.113	0.020	0.0035	2.50E-08	27.495
Reaction time	rs4891744	C	T	0.355	0.014	0.0023	2.14E-09	31.507
Reaction time	rs7351050	A	G	0.162	-0.018	0.0030	4.64E-09	31.013
Reaction time	rs786410	C	C	0.309	0.013	0.0024	3.25E-08	26.988
Reaction time	rs13028903	T	C	0.453	0.019	0.0022	4.15E-18	66.740
Reaction time	rs13027159	G	G	0.260	-0.015	0.0025	1.36E-09	32.311
Reaction time	rs10168817	G	G	0.493	-0.015	0.0022	1.86E-11	39.812
Reaction time	rs16822665	T	C	0.304	-0.015	0.0024	1.87E-10	35.857
Reaction time	rs79177087	T	G	0.196	0.018	0.0028	1.78E-10	35.877
Reaction time	rs994280	G	A	0.341	0.016	0.0023	1.30E-11	39.942
Reaction time	rs6073984	G	A	0.138	-0.019	0.0032	2.85E-09	30.925
Reaction time	rs1115535	C	T	0.275	0.014	0.0025	1.44E-08	28.220
Reaction time	rs6015073	C	C	0.492	-0.013	0.0022	6.36E-09	29.490
Reaction time	rs6062238	C	T	0.317	0.014	0.0024	3.62E-09	30.874
Reaction time	rs874356	A	A	0.405	-0.013	0.0023	4.88E-09	30.333

Reaction time	rs1032501	C	G	0.391	0.013	0.0023	3.03E-08	26.954
Reaction time	rs11719795	T	T	0.295	0.015	0.0024	6.05E-10	33.718
Reaction time	rs4855621	A	G	0.125	-0.018	0.0033	3.61E-08	26.504
Reaction time	rs1231	T	A	0.294	0.014	0.0024	2.29E-09	31.303
Reaction time	rs7684366	T	T	0.220	-0.016	0.0027	2.36E-09	31.392
Reaction time	rs293058	T	C	0.248	-0.015	0.0026	6.35E-09	29.627
Reaction time	rs77917390	A	T	0.099	-0.022	0.0037	3.22E-09	31.548
Reaction time	rs56335290	A	C	0.222	0.019	0.0027	3.62E-12	42.563
Reaction time	rs6870103	G	G	0.450	0.016	0.0022	4.99E-13	46.001
Reaction time	rs74704950	A	G	0.068	0.024	0.0044	3.99E-08	26.424
Reaction time	rs9400241	C	C	0.286	-0.017	0.0025	1.24E-11	41.060
Reaction time	rs6937866	A	G	0.287	-0.015	0.0024	2.51E-10	35.131
Reaction time	rs2040879	T	T	0.174	0.018	0.0029	1.15E-09	32.761
Reaction time	rs7783359	A	T	0.352	0.013	0.0023	1.88E-08	27.743
Reaction time	rs74810647	G	T	0.088	0.022	0.0040	3.34E-08	27.557
Reaction time	rs4875427	G	G	0.322	0.016	0.0024	3.91E-11	38.907
Reaction time	rs10875379	A	C	0.350	0.014	0.0023	4.58E-09	30.158
Reaction time	rs2604268	C	C	0.263	0.018	0.0025	2.30E-13	47.268
Reaction time	rs10125715	T	A	0.262	0.016	0.0025	1.24E-10	36.404

Notes: MR, Mendelian randomization; SNP, single nucleotide polymorphisms; MAF, minor allele frequency; Beta, estimate coefficient; SE, standard error of coefficient estimate.

Supplementary Table S9. Reverse MR analysis for the significant results of mediation MR analyses.

Causal effects were estimated using five two-sample MR methods (IVW, MR Egger, weighted median, weighted mode, and simple mode).

Exposure	Outcome	IVs N	IVW					MR Egger					Weighted Median					Weighted Mode					Simple Mode				
			Estimate (β)	SE	*P* value	95% lower CI	95% upper CI	Estimate (β)	SE	*P* value	95% lower CI	95% upper CI	Estimate (β)	SE	*P* value	95% lower CI	95% upper CI	Estimate (β)	SE	*P* value	95% lower CI	95% upper CI	Estimate (β)	SE	*P* value	95% lower CI	95% upper CI
Visual memory	Fasting insulin	7	0.0131	0.0851	0.8778	-0.1537	0.1799	0.1867	0.8785	0.8401	-1.5352	1.9085	0.0378	0.1089	0.7285	-0.1756	0.2512	0.0719	0.1486	0.6457	-0.2194	0.3632	0.0282	0.1647	0.8698	-0.2947	0.3511
Visual memory	HOMA-IR	7	0.0015	0.0910	0.9867	-0.1768	0.1799	0.0175	0.9460	0.9860	-1.8366	1.8715	-0.0140	0.1111	0.8998	-0.2317	0.2037	-0.0529	0.1521	0.7400	-0.3509	0.2452	-0.0617	0.1488	0.6930	-0.3534	0.2300
Reaction time	Proinsulin	19	0.0138	0.1196	0.9084	-0.2207	0.2482	0.3482	0.8200	0.6764	-1.2590	1.9553	0.0756	0.1691	0.6548	-0.2559	0.4072	0.1494	0.2516	0.5601	-0.3437	0.6425	0.1622	0.3041	0.6002	-0.4338	0.7583
SA of caudal middle frontal	Fasting insulin	6	0.0002	0.0001	0.0429	0.0000	0.0003	0.0002	0.0003	0.5445	-0.0004	0.0009	0.0002	0.0001	0.1097	0.0000	0.0004	0.0002	0.0002	0.3269	-0.0001	0.0005	0.0002	0.0002	0.3678	-0.0002	0.0005
SA of lateral orbitofrontal	Fasting insulin	5	0.0000	0.0002	0.9182	-0.0004	0.0003	-0.0019	0.0010	0.1574	-0.0038	0.0001	0.0000	0.0001	0.7305	-0.0003	0.0002	0.0000	0.0002	0.8229	-0.0004	0.0003	0.0000	0.0002	0.8709	-0.0004	0.0003
SA of medial orbitofrontal	Fasting insulin	2	-0.0006	0.0003	0.0258	-0.0012	-0.0001	NA	NA	NA	NA	NA	NA	NA	NA	NA	NA	NA	NA	NA	NA	NA	NA	NA	NA	NA	NA
SA of precentral	Fasting insulin	3	0.0000	0.0000	0.7283	-0.0001	0.0001	0.0000	0.0001	0.9092	-0.0001	0.0001	0.0000	0.0000	0.7414	-0.0001	0.0001	0.0000	0.0000	0.8045	-0.0001	0.0001	0.0000	0.0001	0.7407	-0.0001	0.0002
SA of insula	Fasting insulin	5	-0.0001	0.0002	0.5285	-0.0004	0.0002	0.0001	0.0006	0.8132	-0.0010	0.0013	0.0000	0.0002	0.8684	-0.0004	0.0004	0.0002	0.0003	0.6211	-0.0005	0.0008	0.0002	0.0003	0.6306	-0.0005	0.0008
SA of precentral	HOMA-IR	3	0.0000	0.0001	0.9072	-0.0001	0.0001	0.0000	0.0000	0.8797	-0.0001	0.0001	0.0000	0.0000	0.8960	-0.0001	0.0001	0.0000	0.0001	0.8659	-0.0001	0.0001	0.0000	0.0000	0.8841	-0.0001	0.0001
SA of insula	HOMA-IR	5	-0.0001	0.0002	0.4289	-0.0004	0.0002	0.0002	0.0005	0.6917	-0.0008	0.0012	-0.0001	0.0002	0.6482	-0.0005	0.0003	-0.0004	0.0003	0.2769	-0.0010	0.0002	0.0001	0.0003	0.7548	-0.0005	0.0007
SA of rostral middle frontal	Proinsulin	7	-0.0002	0.0001	0.0884	-0.0005	0.0000	0.0005	0.0010	0.6611	-0.0014	0.0023	-0.0002	0.0002	0.1252	-0.0006	0.0001	-0.0003	0.0002	0.3017	-0.0008	0.0002	-0.0003	0.0002	0.2954	-0.0008	0.0002
SA of cuneus	Proinsulin	7	0.0000	0.0005	0.9256	-0.0009	0.0010	0.0012	0.0021	0.5887	-0.0028	0.0052	-0.0001	0.0005	0.8441	-0.0011	0.0009	-0.0002	0.0007	0.8087	-0.0016	0.0012	0.0001	0.0008	0.9510	-0.0016	0.0017
TH of rostral anterior cingulat	Fasting insulin	3	-0.1958	0.2954	0.5075	-0.7748	0.3833	-1.7432	2.2681	0.5828	-6.1887	2.7022	-0.0203	0.2881	0.9439	-0.5849	0.5444	0.0964	0.4009	0.8324	-0.6894	0.8822	0.1017	0.3687	0.8086	-0.6210	0.8244
TH of parahippocampal	Fasting insulin	16	-0.0341	0.0893	0.7027	-0.2090	0.1409	-0.2964	0.3318	0.3868	-0.9466	0.3539	-0.0663	0.0929	0.4751	-0.2484	0.1157	-0.0679	0.1485	0.6541	-0.3590	0.2232	0.0009	0.1702	0.9959	-0.3328	0.3346
TH of pericalcarine	Fasting insulin	9	0.0467	0.2553	0.8548	-0.4537	0.5471	2.4592	2.5936	0.3746	-2.6242	7.5425	0.0897	0.3069	0.7701	-0.5119	0.6912	0.6725	0.6160	0.3067	-0.5348	1.8798	0.6725	0.5696	0.2717	-0.4440	1.7890
TH of rostral anterior cingulat	HOMA-IR	3	-0.1960	0.3107	0.5282	-0.8050	0.4130	-2.2552	2.0268	0.4661	-6.2278	1.7173	-0.1400	0.3059	0.6473	-0.7395	0.4596	-0.0617	0.4037	0.8926	-0.8530	0.7297	-0.0418	0.4188	0.9296	-0.8628	0.7791
TH of caudal anterior cingulat	Proinsulin	2	0.9433	0.6223	0.1296	-0.2764	2.1630	NA	NA	NA	NA	NA	NA	NA	NA	NA	NA	NA	NA	NA	NA	NA	NA	NA	NA	NA	NA
TH of bankssts	Proinsulin	9	-0.5439	0.4514	0.2282	-1.4287	0.3409	-1.1092	1.4112	0.4577	-3.8752	1.6568	-0.3193	0.5902	0.5885	-1.4760	0.8375	-0.3146	0.8373	0.7169	-1.9556	1.3265	-0.1704	0.9389	0.8605	-2.0107	1.6698
TH of parahippocampal	Proinsulin	16	-0.2608	0.1607	0.1048	-0.5758	0.0543	-0.8489	0.5511	0.1458	-1.9290	0.2313	-0.3171	0.2281	0.1646	-0.7642	0.1300	-0.4564	0.3884	0.2582	-1.2176	0.3048	-0.3682	0.3980	0.3695	-1.1483	0.4119
TH of posterior cingulate	Proinsulin	5	0.2439	0.6468	0.7061	-1.0238	1.5116	0.2075	3.1605	0.9518	-5.9871	6.4021	0.0017	0.7934	0.9983	-1.5535	1.5568	-0.0731	1.0307	0.9468	-2.0934	1.9471	0.0227	1.1149	0.9847	-2.1624	2.2078
Reaction time	SA of rostral middle fronta	31	-66.9115	49.9717	0.1806	-164.8560	31.0331	-184.4291	289.3242	0.5288	-751.5044	382.6463	-124.7233	68.1623	0.0673	-258.3214	8.8747	-168.8079	127.7085	0.1962	-419.1166	81.5008	-154.9526	144.2461	0.2913	-437.6749	127.7697

Notes: MR, Mendelian randomization; IVW, inverse variance weighted; IVs, instrumental variables; β, estimate coefficient; SE, standard error of coefficient estimate; CI, confidence interval; HOMA-B, homeostasis model assessment beta-cell function; HOMA-IR, homeostasis model assessment insulin resistance; SA, surficial area; TH, thickness.

Supplementary Table S10. Heterogeneity and pleiotropy assessment for the reverse MR analyses.

Exposure	Outcome	Cochrane's Q test (MR Egger)		Cochrane's Q test (IVW)		MR-PRESSO global test		MR-Egger intercept		
		Q' test	P value	Q' test	P value	RSS_{obs}	P value	Egger_intercept	SE	P value
Visual memory	Fasting insulin	3.407	0.638	3.446	0.751	4.852	0.767	-0.003	0.013	0.850
Visual memory	HOMA-IR	2.227	0.817	2.227	0.898	3.082	0.891	0.000	0.014	0.987
Reaction time	Proinsulin	15.286	0.575	15.456	0.630	17.082	0.655	-0.005	0.012	0.685
SA of caudal middle frontal	Fasting insulin	3.891	0.421	3.923	0.561	6.459	0.561	-0.001	0.005	0.867
SA of lateral orbitofrontal	Fasting insulin	4.847	0.183	10.529	0.032	15.139	0.076	0.022	0.012	0.157
SA of medial orbitofrontal	Fasting insulin	NA	NA	0.306	0.580	NA	NA	NA	NA	NA
SA of precentral	Fasting insulin	0.001	0.978	0.017	0.992	NA	NA	0.000	0.003	0.919
SA of insula	Fasting insulin	5.077	0.166	5.452	0.244	8.692	0.270	-0.003	0.006	0.670
SA of precentral	HOMA-IR	0.038	0.845	0.044	0.978	NA	NA	0.000	0.003	0.950
SA of insula	HOMA-IR	3.369	0.338	3.938	0.414	6.270	0.440	-0.004	0.005	0.528
SA of rostral middle frontal	Proinsulin	1.036	0.960	1.541	0.957	2.135	0.961	-0.015	0.021	0.509
SA of cuneus	Proinsulin	8.707	0.121	9.279	0.158	12.379	0.189	-0.011	0.019	0.591
TH of rostral anterior cingulat	Fasting insulin	2.907	0.088	4.292	0.117	NA	NA	0.016	0.024	0.615
TH of parahippocampal	Fasting insulin	31.275	0.005	32.783	0.005	37.206	0.008	0.004	0.004	0.425
TH of pericalcarine	Fasting insulin	11.537	0.117	12.977	0.113	16.575	0.101	-0.012	0.013	0.381
TH of rostral anterior cingulat	HOMA-IR	2.047	0.152	4.210	0.122	NA	NA	0.022	0.021	0.491
TH of caudal anterior cingulat	Proinsulin	NA	NA	0.745	0.388	NA	NA	NA	NA	NA
TH of bankssts	Proinsulin	4.824	0.681	5.002	0.757	6.280	0.756	0.004	0.010	0.685
TH of parahippocampal	Proinsulin	12.249	0.586	13.494	0.564	15.830	0.556	0.008	0.007	0.283
TH of posterior cingulate	Proinsulin	2.231	0.526	2.231	0.693	3.412	0.708	0.000	0.020	0.991
Reaction time	SA of rostral middle fronta	17.705	0.950	17.875	0.961	19.051	0.948	1.863	4.518	0.683

Notes: MR, Mendelian randomization; IVW, inverse variance weighted; MR-PRESSO, Mendelian randomization pleiotropy residual sum and outlier; RSSobs, observed residual sum of squares; SE, standard error of coefficient estimate; HOMA-B, homeostasis model assessment beta-cell function; HOMA-IR, homeostasis model assessment insulin resistance; SA, surficial area; TH,
